# Genome diversity of Chinese indigenous chicken and the selective signatures in Chinese gamecock chicken

**DOI:** 10.1038/s41598-020-71421-z

**Published:** 2020-09-03

**Authors:** Wei Luo, Chenglong Luo, Meng Wang, Lijin Guo, Xiaolan Chen, Zhenhui Li, Ming Zheng, Bello Semiu Folaniyi, Wen Luo, Dingming Shu, Linliang Song, Meixia Fang, Xiquan Zhang, Hao Qu, Qinghua Nie

**Affiliations:** 1 Department of Animal Genetics, Breeding and Reproduction, College of Animal Science, Guangzhou, 510642 Guangdong China; 2 State Key Laboratory of Livestock and Poultry Breeding and Guangdong Key Laboratory of Animal Breeding and Nutrition, Institute of Animal Science, Guangdong Academy of Agricultural Sciences, Guangzhou, 510640 China; 3 Guangdong Provincial Key Lab of Agro-Animal Genomics and Molecular Breeding and Key Lab of Chicken Genetics, Breeding and Reproduction, Ministry of Agriculture, Guangzhou, 510642 Guangdong China; 4 Novogene Bioinformatics Institute, Beijing, China; 5 Institute of Laboratory Animals, Jinan University, Guangzhou, Guangdong China

**Keywords:** Computational biology and bioinformatics, Evolution, Genetics, Zoology

## Abstract

Gamecock chickens are one of the earliest recorded birds in China, and have accumulated some unique morphological and behavioral signatures such as large body size, muscularity and aggressive behavior, whereby being excellent breeding materials and a good model for studying bird muscular development and behavior. In this study, we sequenced 126 chicken genomes from 19 populations, including four commercial chicken breeds that are commonly farmed in China, 13 nationwide Chinese typical indigenous chicken breeds (including two Chinese gamecock breeds), one red jungle fowl from Guangxi Province of China and three gamecock chickens from Laos. Combined with 31 published chicken genomes from three populations, a comparative genomics analysis was performed across 157 chickens. We found a severe confounding effect on potential cold adaptation exerted by introgression from commercial chickens into Chinese indigenous chickens, and argued that the genetic introgression from commercial chickens into indigenous chickens should be seriously considered for identifying selection footprint in indigenous chickens. LX gamecock chickens might have played a core role in recent breeding and conservation of other Chinese gamecock chickens. Importantly, *AGMO* (*Alkylglycerol monooxygenase*) and *CPZ* (*Carboxypeptidase Z*) might be crucial for determining the behavioral pattern of gamecock chickens, while *ISPD* (*Isoprenoid synthase domain containing*) might be essential for the muscularity of gamecock chickens. Our results can further the understanding of the evolution of Chinese gamecock chickens, especially the genetic basis of gamecock chickens revealed here was valuable for us to better understand the mechanisms underlying the behavioral pattern and the muscular development in chicken.

## Introduction

China as a possible origin of domestic chickens and a vast country with abundant diversities in geography and culture^1,2^, it has accumulated the most abundant genetic resources in Chinese indigenous chickens under extensive natural and artificial selections, with considerable genetic variations and phenotypic diversity in terms of morphology and physiology^3–8^. A comprehensive and deep understanding of the genome diversity of the Chinese indigenous breeds could reveal the population dynamics of the breeds, providing a theoretical basis for facilitating conservations and breeding programs. Also, this can provide us a good opportunity in understanding the interplay between genetic variations and phenotypic diversities in chicken.

Chicken has been the main source of protein in the human diet but at the onset of thousands of years of chicken explorations, symbolic and social domains such as cockfighting are ahead of economic explorations^9^. Chinese gamecock chickens are one of the earliest recorded birds in China which can be dated back to 2700 bc and characterized by their special utility for cockfighting^3,10^. Accompanied by a long artificial selection, Chinese gamecock chickens have accumulated some unique morphological and behavioral signatures such as small comb size, large body size, muscularity and aggressive behavior^3^, whereby being excellent breeding materials and a good model for studying bird behavior.

In a previous pioneering study concerning selective signatures in BN gamecock chickens, Guo et al. highlighted several numbers of candidate selective genes underlying signatures of gamecock chickens^11^, such as organ development-related genes: *CBFB* (*Core-binding factor beta subunit*), *GRHL3* (*Grainyhead like transcription factor 3*), *Gli3* (*GLI family zinc finger 3*), *PTCH1* (*Patched 1*) and *EFNA5* (*EphrinA5*), aggressive behavior related genes: *BDNF* (*Brain-derived neurotrophic factor*), *NTS* (*Neurotensin*) and *GNAO1* (*G protein subunit alpha o1*), energy metabolism-related genes: *RICTOR* (*RPTOR independent companion of MTOR complex 2*) and *SDHB* (*Succinate dehydrogenase complex iron-sulfur subunit B*). However, this study is likely to be biased by genetic drift and confounded by potential artificial selection undergone in Chinese indigenous and commercial chickens, as this study just concerned one gamecock breed, and did not fully consider potential introgression from Chinese indigenous and commercial chickens into Chinese gamecock chickens because an introgression from commercial chickens into indigenous chickens seems common^7,8,12^. Incorporating more gamecock breeds and taking Chinese indigenous and commercial chickens into account in a subsequent whole genome re-sequencing study can help us totally reveal the pivotal variants/genes underlying signatures in gamecock chickens.

Also, natural selections especially the extreme environments have proved to be important driving forces in shaping genome diversity of animals (pig, cattle, sheep and horse) since their domestications^13–16^. Chicken has spread worldwide since possibly domesticated in Southeast Asia and Southwest China before 2000–6000 bc^1,17^, and evolutionarily adapted to a variety of local environments, such as high altitude, aridness and stressful African conditions (e.g., disease resistance, poor nutrition, oxidative and heat stresses)^4,18,19^. Similarly, the Chinese indigenous chickens from high-latitude zones have also evolutionarily adapted to the cold winter^3^, compared with the wild ancestor red jungle fowls^20^, which inhabit in tropical areas. Distinct from commercial chickens, Chinese indigenous chickens are less intensively selected^8^, decreasing the possibility of those genomic footprints left by natural selection are to be obscured by strong artificial selection. Aside from a potential introgression from commercial chickens into indigenous chickens, the Chinese indigenous chickens from low-latitude to high-latitude zones are likely to be a good model for exploring the genetic mechanisms underlying rapid adaptation to cold weather in birds within a short period of time.

In this research, we sampled and whole-genome re-sequenced 126 chicken individuals, which included four typical commercial chicken breeds that are commonly farmed in China, two Chinese gamecock breeds, another 11 Chinese nationwide canonical indigenous chicken breeds, one red jungle fowl population from Guangxi Province of China, and three gamecock chickens from Laos (Note S1; Table S1). Combined with the genome sequencing data of 31 chickens (Tibetan chicken, BN gamecock breed, Yunnan village chicken, and Red jungle fowls) that were previously published^4^, these together allowed us to get a deeper and more comprehensive understanding of the genomic variants/genes underlying the signatures in gamecock chickens, and evaluate the potential genomic footprints left by cold adaptation.

## Results and discussion

### Sequencing and genomic variant

In the present study, we whole-genome re-sequenced a total of 126 chicken samples, generating a panel of clean data ranging from 9.5 to 60.2 billion base pairs (bp), corresponding to genome coverage ranging from 7.0**× **to 48.9**× **(Table S2). Except for the Sample Laos03 (mapping rate = 94.85%), the mapping rate of the other 125 individuals was greater than 97%. The ratio of the genome covered with at least one sequencing base ranged from 89.1 to 93.5%, while covered with at least four sequencing bases ranged from 75.0 to 91.60%.

After incorporating the sequencing data of another public 31 Chinese indigenous chickens, we identified a total of 17,375,012 raw SNPs and 1,726,022 raw InDels via SAMtools, and a total of 43,643,339 raw SNPs and 4,326,184 InDels via GATK pipeline, in which a total of 17,349,501 SNPs and 1,673,029 InDels shared by both SAMtools and GATK pipelines were further identified. Following the filtration criteria in “Materials and methods” (2.2), a total of 10,119,242 genome-wide population SNPs (Table S3; Fig. S1) and 837,787 genome-wide population InDels (Table S4; Fig. S2) were obtained. For this set of 10,119,242 genome-wide population SNPs, which composed of 9,463,354 known and 655,888 novel SNPs, and included 9,794,983 autosomal SNPs. Compared with previous whole-genome resequencing studies in Chinese chickens^4,6^, the number of novelSNPs and InDels identified here is relatively smaller, and this is probably because we employed two pipelines together to call the variants and large non-uniformity in terms of sequencing depth existed in 157 samples. For the SNPs abundance in all 22 populations, RJF harbored the highest in terms of both total and novel SNPs. Apart from RJF, in the populations sequenced in this study, TLF gamecock chickens exhibited the highest abundance in terms of both total and novel SNPs, while LH exhibited the lowest. For the 837,787 genome-wide population InDels, it had 382,059 insertions and 455,728 deletions. The abundance pattern across 22 populations in terms of InDels was similar to that in SNPs.

### Genome-wide nucleotide diversity and heterozygosity, linkage disequilibrium

**Figure 1:**
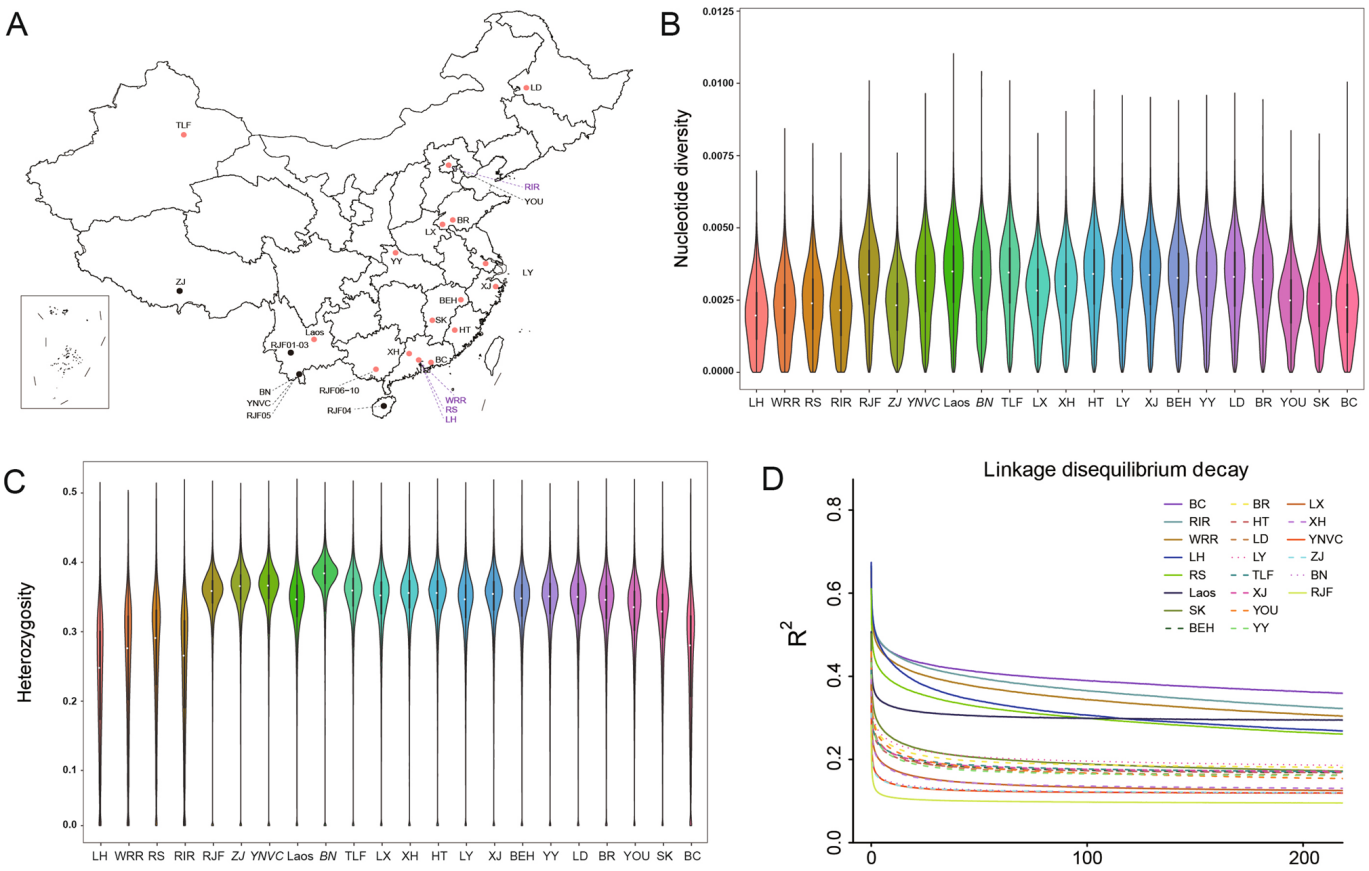
Geographical distribution, genome diversity and LD decay of 22 chicken populations analyzed in this study. (**A**) Origin of samples used in the present study. In this Chinese geographical map (generated by ArcGIS version 10.7; the corresponding Shapefile is downloaded from https://www.resdc.cn/data.aspx?DATAID=201), the red dot represents the sampled population in this study, while the black dot represents the population previously sequenced. (**B**) Genome nucleotide diversity of 22 populations, for which it was calculated with a window size of 40 Kb and a step size of 20 Kb. The population with italic font indicates the previously sequenced. (**C**) Linkage disequilibrium (LD) decay of 22 populations, denoted with one line for each population. (**D**) Genome heterozygosity of 22 populations. The calculation window and step size are the same as those in genome nucleotide diversity above.

Among all 22 populations, we observed the lowest genome-wide $\pi$ in three commercial populations, lowest in LH chickens ($pop\_\pi$ = 0.00199), followed by RIR chickens ($pop\_\pi$ = 0.00216) and WRR chickens ($pop\_\pi$ = 0.00223) (Fig. 1B). While among the Chinese indigenous chickens, the populations with Muffs and Beard phenotype, including BC chicken ($pop\_\pi$ = 0.00225), SK chickens ($pop\_\pi$ = 0.00234) and YOU chickens ($pop\_\pi$ = 0.00246) harbored the lowest genome-wide $\pi$. Across the three Chinese gamecock populations, we observed the highest genome-wide $\pi$ in TLF chickens ($pop\_\pi$ = 0.00333), followed by BN chickens ($pop\_\pi$ = 0.00316) and LX chickens ($pop\_\pi$ = 0.00276). Similar to the results in genome-wide nucleotide diversity, we also observed the lowest population heterozygosity (*pop_He*) in three commercial populations, lowest in LH chickens (*pop_He* = 0.2114), followed by RIR chickens (*pop_Hp* = 0.2257) and WRR chickens (*pop_He* = 0.2337) (Fig. 1C). While the population with muffs and beard phenotype, including BC chickens (*pop-He* = 0.2376), SK chickens (*pop-He* = 0.2929) and YOU chickens (*pop-He* = 0.3010), harbored the lowest *pop-He* in Chinese indigenous chickens. Among gamecock chickens, there were distinct results of $pop\_\pi$, BN chickens (*pop_Hp* = 0.3555) but not TLF chickens (*pop_Hp* = 0.3299), harbored the highest *pop_Hp*. Also, we observed the highest level of LD in BC chickens, followed by four commercial populations and SK chickens, while the lowest was recorded in RJF chickens. Besides, the three Chinese gamecock chickens showed a low level in LD, highest by TLF chickens, followed by LX and BN chickens (Fig. 1D).

Except three breeds (BC, SK, and YOU) with muffs and beard phenotype, the other Chinese indigenous chickens have undergone much less intensive artificial selection compared with commercial chickens, which are basically consistent with previous findings^4,7,8^. Surprisingly, as one of the earliest recorded chicken populations in China^3^, Chinese gamecock chickens have been supposed to be under strong artificial selection since they have been consistently selected for cockfighting. But in the present study, similar to most Chinese indigenous chickens, the gamecock chickens also harbored relatively low levels in terms of nucleotide diversity and LD, and high level of heterozygosity, suggestive to a limited number of genes possibly underlying the signatures observed in gamecock chickens.

### Genome-wide genetic differentiation

We observed the highest levels (mean weighted Fst > 0.2) of genetic differentiation between each commercial population and Chinese indigenous chickens, especially between LH chickens and BC chickens (Weighted Fst = 0.4365) (Table 1). Between the Chinese indigenous chicken populations, a higher level of genetic differentiation occurred between the three populations with Muffs and Beard phenotype (BC, SK, and YOU chickens) and others (Table 1), which were all higher than that of RJF chickens against other Chinese indigenous chickens (P < 0.01), suggestive of the driving force of this unique external trait in shaping the genomic variation pattern. In particular, the weighted Fst values (0.255 ± 0.07) between BC chickens and other Chinese indigenous chickens could be comparable to those of four commercial populations against other Chinese indigenous chickens. BC as a Chinese indigenous chicken breed originated from Guangdong Province, which is adjacent to the habitat of RJF (Fig. 1A). This does not totally support the argument given by Nie et al.^7,8^, that a critical role in shaping the genomic variation within Eurasia continent chicken breeds might have been played by isolation based on distance. We propose strong artificial selection, together with isolation by distance are the main driving forces in shaping chicken genomic variation.

**Table 1:** Genome-wide genetic differentiation between each population.

Populations	Weighted Fst																					
	RJF	BN	LX	TLF	YNVC	Laos	ZJ	XH	BC	HT	SK	BEH	XJ	LY	BR	YY	YOU	LD	RIR	RS	WRR	LH
RJF		0.060	0.107	0.080	0.049	0.057	0.059	0.091	0.223	0.080	0.155	0.093	0.085	0.104	0.105	0.091	0.138	0.093	0.236	0.205	0.226	0.247
BN	0.060		0.061	0.023	0.023	0.007	0.050	0.056	0.191	0.052	0.128	0.068	0.056	0.074	0.082	0.067	0.111	0.070	0.216	0.185	0.207	0.250
LX	0.107	0.061		0.046	0.071	0.078	0.088	0.090	0.227	0.075	0.137	0.078	0.076	0.103	0.086	0.066	0.114	0.071	0.236	0.209	0.234	0.302
TLF	0.080	0.023	0.046		0.041	0.036	0.065	0.067	0.216	0.060	0.134	0.072	0.064	0.085	0.085	0.067	0.113	0.072	0.236	0.205	0.230	0.289
YNVC	0.049	0.023	0.071	0.041		0.017	0.032	0.051	0.195	0.043	0.121	0.057	0.048	0.068	0.072	0.054	0.104	0.060	0.214	0.180	0.202	0.247
Laos	0.057	0.007	0.078	0.036	0.017		0.047	0.061	0.234	0.055	0.143	0.074	0.062	0.082	0.091	0.071	0.125	0.076	0.264	0.221	0.251	0.304
ZJ	0.059	0.050	0.088	0.065	0.032	0.047		0.075	0.213	0.059	0.135	0.072	0.062	0.077	0.076	0.064	0.113	0.062	0.212	0.179	0.201	0.224
XH	0.091	0.056	0.090	0.067	0.051	0.061	0.075		0.212	0.060	0.135	0.074	0.065	0.088	0.090	0.072	0.118	0.079	0.229	0.201	0.225	0.283
BC	0.223	0.191	0.227	0.216	0.195	0.234	0.213	0.212		0.210	0.275	0.223	0.214	0.232	0.237	0.220	0.257	0.225	0.385	0.350	0.376	0.436
HT	0.080	0.052	0.075	0.060	0.043	0.055	0.059	0.060	0.210		0.117	0.049	0.043	0.066	0.064	0.045	0.097	0.051	0.212	0.181	0.207	0.280
SK	0.155	0.128	0.137	0.134	0.121	0.143	0.135	0.135	0.275	0.117		0.120	0.120	0.148	0.128	0.110	0.160	0.117	0.280	0.251	0.275	0.347
BEH	0.093	0.068	0.078	0.072	0.057	0.074	0.072	0.074	0.223	0.049	0.120		0.052	0.084	0.067	0.043	0.100	0.052	0.227	0.198	0.223	0.304
XJ	0.085	0.056	0.076	0.064	0.048	0.062	0.062	0.065	0.214	0.043	0.120	0.052		0.064	0.060	0.045	0.094	0.048	0.205	0.179	0.204	0.279
LY	0.104	0.074	0.103	0.085	0.068	0.082	0.077	0.088	0.232	0.066	0.148	0.084	0.064		0.080	0.073	0.115	0.070	0.218	0.174	0.189	0.276
BR	0.105	0.082	0.086	0.085	0.072	0.091	0.076	0.090	0.237	0.064	0.128	0.067	0.060	0.080		0.051	0.101	0.045	0.201	0.187	0.210	0.283
YY	0.091	0.067	0.066	0.067	0.054	0.071	0.064	0.072	0.220	0.045	0.110	0.043	0.045	0.073	0.051		0.088	0.034	0.212	0.184	0.210	0.296
YOU	0.138	0.111	0.114	0.113	0.104	0.125	0.113	0.118	0.257	0.097	0.160	0.100	0.094	0.115	0.101	0.088		0.087	0.246	0.218	0.242	0.316
LD	0.093	0.070	0.071	0.072	0.060	0.076	0.062	0.079	0.225	0.051	0.117	0.052	0.048	0.070	0.045	0.034	0.087		0.199	0.176	0.203	0.266
RIR	0.236	0.216	0.236	0.236	0.214	0.264	0.212	0.229	0.385	0.212	0.280	0.227	0.205	0.218	0.201	0.212	0.246	0.199		0.327	0.353	0.415
RS	0.205	0.185	0.209	0.205	0.180	0.221	0.179	0.201	0.350	0.181	0.251	0.198	0.179	0.174	0.187	0.184	0.218	0.176	0.327		0.284	0.368
WRR	0.226	0.207	0.234	0.230	0.202	0.251	0.201	0.225	0.376	0.207	0.275	0.223	0.204	0.189	0.210	0.210	0.242	0.203	0.353	0.284		0.392
LH	0.247	0.250	0.302	0.289	0.247	0.304	0.224	0.283	0.436	0.280	0.347	0.304	0.279	0.276	0.283	0.296	0.316	0.266	0.415	0.368	0.392	
Mean Weighted Fst	0.123 ± 0.06	0.097 ± 0.07	0.122 ± 0.07	0.109 ± 0.08	0.093 ± 0.07	0.112 ± 0.09	0.103 ± 0.06	0.115 ± 0.07	0.255 ± 0.07	0.100 ± 0.07	0.168 ± 0.07	0.111 ± 0.07	0.101 ± 0.07	0.118 ± 0.06	0.114 ± 0.07	0.103 ± 0.07	0.146 ± 0.07	0.103 ± 0.07	0.253 ± 0.06	0.222 ± 0.06	0.245 ± 0.06	0.305 ± 0.06

Weir Fst weighted values are used to denote the genetic differentiation.

### Population genetic structure analysis unveiled a high level of admixture across Chinese gamecock chickens

**Figure 2:**
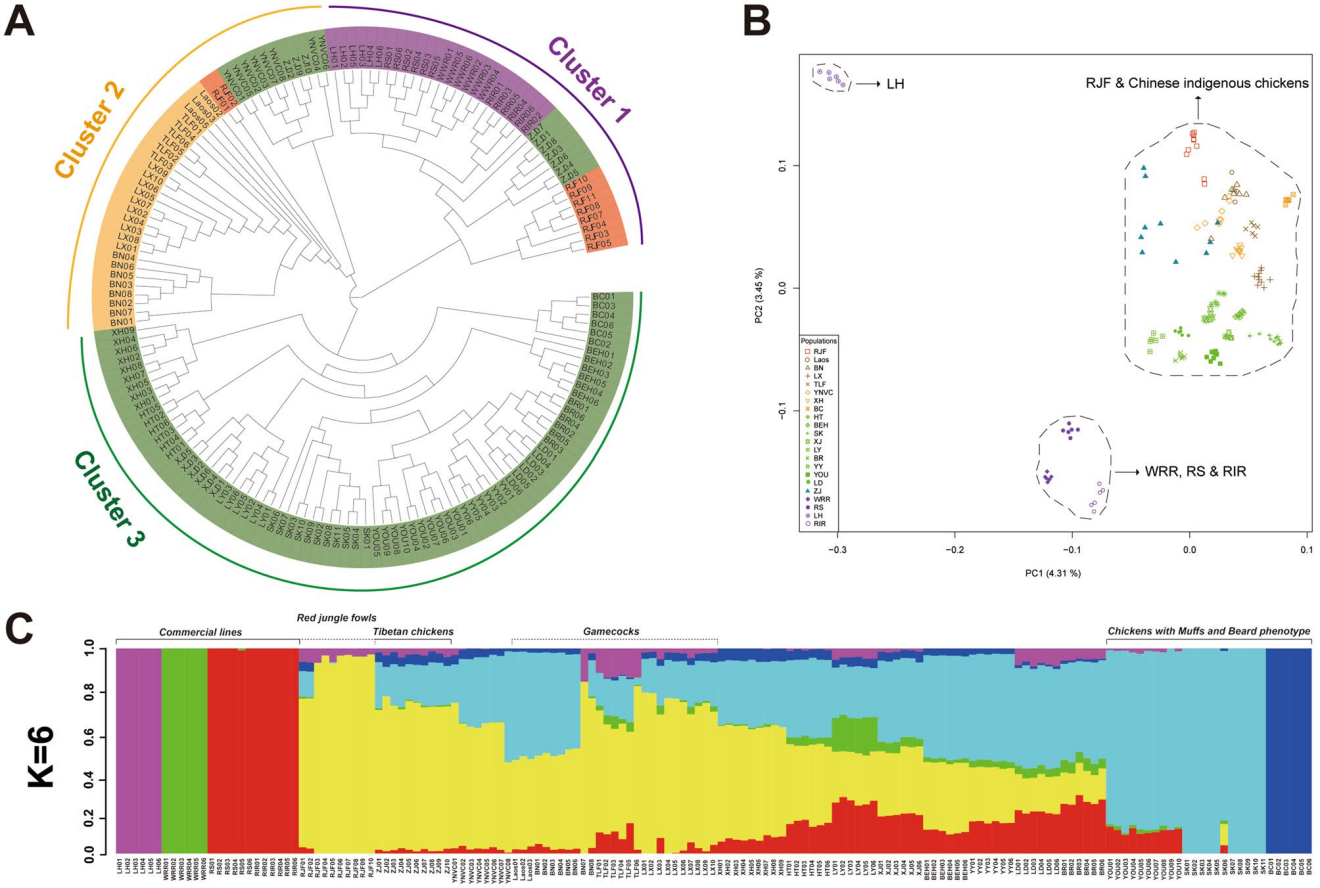
Population structure analyses of 22 chicken populations of this study. (**A**) Neighbor-joining phylogenetic tree of 157 chickens, constructed with TreeBeST version 1.9.2 (https://sourceforge.net/projects/treesoft/files/treebest/1.9.2/) without an outgroup. (**B**) Principal component analysis (PCA), with 4.31% and 3.45% variance explained in PC1 and PC2, respectively. (**C**) Admixture analysis at K = 6 (best assumed genetic group).

According to the neighbor-joining tree, all the 157 chickens from 22 populations could be separated into three clusters (Fig. 2A). Of them, Cluster 1 included four commercial populations, seven ZJ, and eight RJF individuals; Cluster 2 included the populations of four gamecock populations and YNVC chickens, and the individuals of 3 ZJ and 2 RJF, suggestive of a possible same origin of the Chinese gamecock chickens included here; while the remaining 11 Chinese indigenous populations composed the Cluster 3. Consistent with the previous study^4^, RJF and ZJ chickens could be separated into two different clusters in the present study. Except for the populations of RJF, ZJ, YNVC and Laos gamecock chickens, all the left populations could be separated into its own clade.

However, the PCA results could not completely reproduce the phylogenetic relationships. The top two PCs (4.31% and 3.45% variances explained totally, respectively) could separate the four commercial populations from non-commercial populations (Fig. 2B). Especially, the 10 RJF chickens here were genetically classified very near the Chinese indigenous chickens but away from commercial chickens, which is consistent with the study of Wang et al.^2^, and probably due to that these RJF chickens belong to *G. g. spadiceus*. LH as a population harboring the highest level of genetic differentiation with others (Table 1), its unique genetic variation pattern could also be evidenced in the top two PCs being an independent cluster from others. But the four gamecock populations, together with YNVC, 3 ZJ, and 2 RJF chickens could not be well grouped into one cluster as indicated by the above phylogenetic analysis, suggestive of a complex genetic structure of them. Within each population, we observed 3 and 2 outlier samples in ZJ and RJF chickens, which were also revealed in the above phylogenetic analysis. Besides, it was hard to separate the populations of Laos, BN, ZJ and, YNVC from each other, suggestive of potential admixture among them.

To infer the admixture degree across 157 samples, we further performed an unsupervised Admixture analysis, with K run from 2 to 16. We found at K = 2, consistent with the above PCA result (Fig. 2B), genetic divergency first occurred between commercial populations and non-commercial ones. Except for BC and SK (except SK06), a potential widespread genetic introgression from commercial populations to other Chinese indigenous ones was observed across K = 2 to K = 5 (Figure S3), inclusively. As suggested by the cross-validation errors (Figure S4), K = 6 was the best assumed genetic groups in this study. At K = 6 (Fig. 2C), LH, WRR, SK (except SK06) and BC chickens, these four populations could still keep distinct; RS and RIR chickens formed another group; While the last group was mainly represented by RJF chickens. For the gamecock chickens, they genetically appeared to be the admixture of RJF, Chinese indigenous and commercial chickens. Clearly, except for SK (excluding SK06) and BC chickens, we could still observe a potential widespread genetic introgression from commercial chickens to most Chinese indigenous chickens, at K = 6, which agrees with the previous study^8^.

### TreeMix analysis revealed evidence of gene flows from LX gamecock chickens into other gamecock chickens

**Figure 3:**
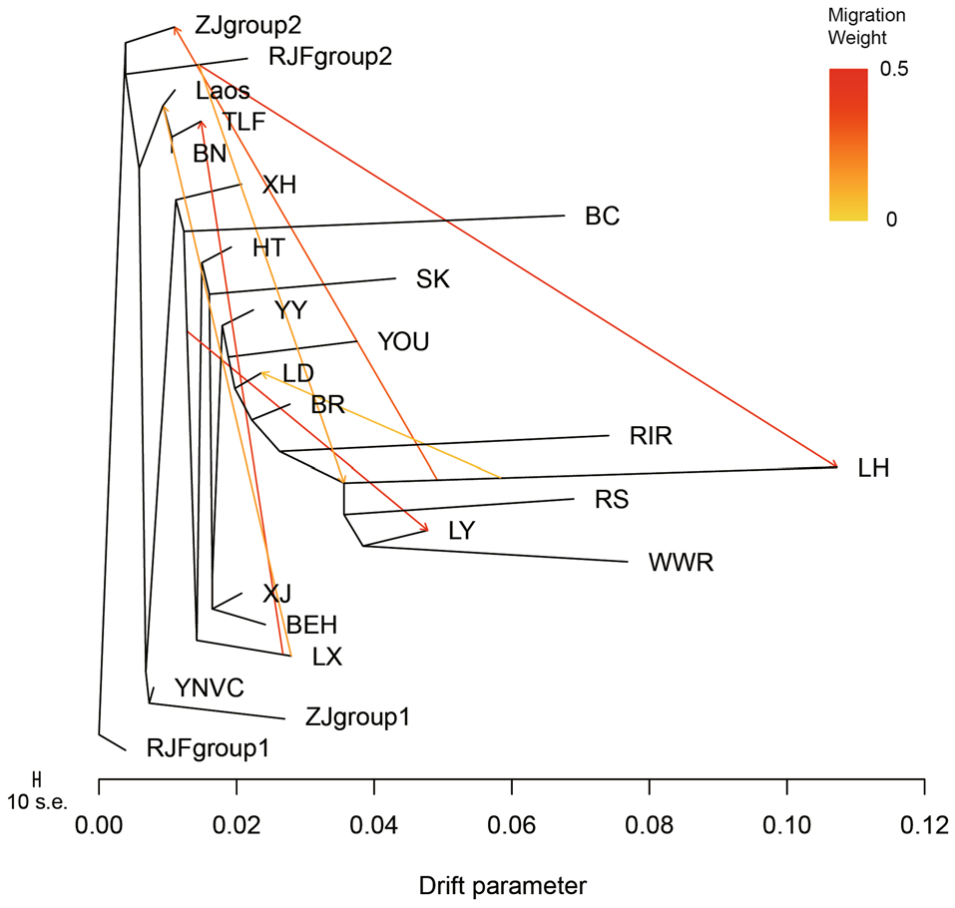
TreeMix analysis of Chinese indigenous and commercial chickens. This maximum likelihood tree explains 92.09% variance after adding seven migrations. Migration weight is given according to the color of the arrows. The scale bar denotes ten times the average standard error of the entries in the sample covariance matrix.

Given that a potential widespread introgression from commercial chickens to most Chinese indigenous breeds has been suggested by above Admixture analysis, and to better understand the historical relationship within the 22 populations, we further employed TreeMix to reconstruct a maximum likelihood (ML) tree, in which it allows both populations split and migration events. We found the inferred migration edges at seven could return the smallest residuals (Figure S5), thus being the best fit for our data. In this ML tree (Fig. 3), two gene flows from LH chickens into two Chinese indigenous breeds, including ZJ and LD chickens could be evidenced, which conformed with the Admixture results of that a potential widespread introgression from commercial chickens into Chinese indigenous chickens. Noticeably, among the three gene flows between Chinese indigenous breeds, two of them both indicated the gene flows from LX gamecock chickens into the other three gamecock breeds. LX gamecock chicken can be dated back to 700 bc^3^, and is one of the earliest documented Chinese indigenous chicken breeds, conferring it more advantages in the utilization of cockfighting. This may together suggest a core role played by LX gamecock chickens in recent breeding and conservation of Chinese indigenous gamecock chickens.

### Severe confounding effect on selective signatures of cold adaptation exerted by genetic introgression from commercial chickens to Chinese indigenous chickens

We observed strong artificial selection that has been undergone in commercial populations here, which exhibited lower nucleotide diversity, lower heterozygosity and higher LD level within populations, and strikingly high genetic differentiation with Chinese indigenous chickens, at the genome-wide level. More importantly, Admixture analysis inferred a potential widespread genetic introgression from commercial chickens into Chinese indigenous chickens except for BC and SK, which can be also partially evidenced. This means any selective signatures especially presented by natural selection to be identified in Chinese indigenous populations will be probably extremely confounded by strong artificial selection undergone in commercial chickens. We argued here that the genetic introgression from commercial chickens into indigenous chickens should be quite seriously considered when identifying selective signatures presented in indigenous chickens, in terms of natural and artificial selections.

**Figure 4:**
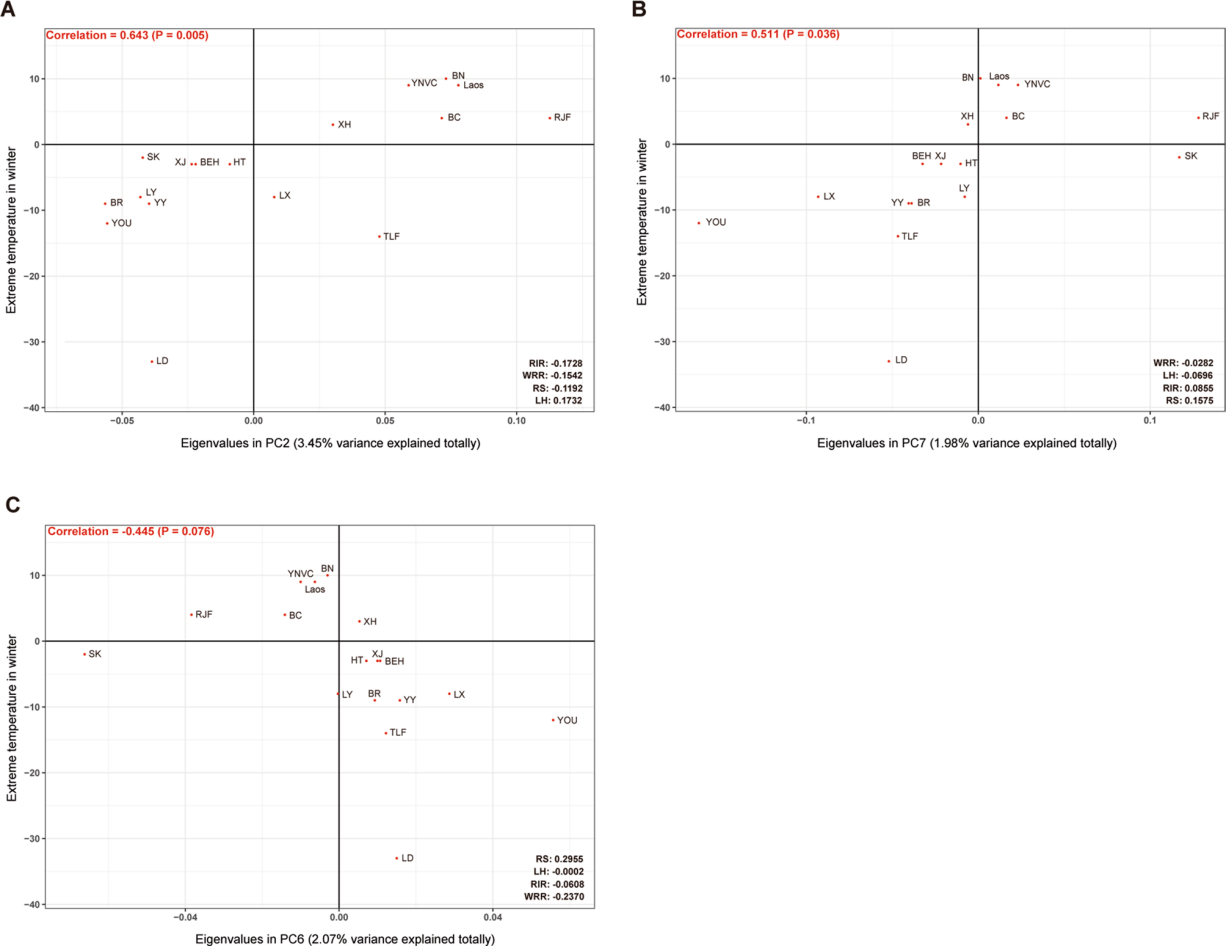
Correlation analyses between the extreme temperature in winter and the eigenvalues of each Chinese indigenous population from PC2 (**A**), PC7 (**B**) and PC6 (**C**). The extreme temperature used here was the statistics from 2017 and 2018, cited from the China Meteorological Administration. The eigenvalues of each population given here were the average within each.

Given an example concerning to potential cold adaptation in Chinese high-latitude indigenous chickens, this confounding effect exerted by genetic introgression from commercial chickens could be observed by performing correlation analysis between the average eigenvalues (population eigenvalues) of each Chinese indigenous population from PC1 to PC10 (Raw data was from above PCA; Table S5) and the corresponding inhibiting extreme temperatures of each population in winter (Table S1), population eigenvalues of the Chinese indigenous in PC2 (3.45% variance explained totally) was found to be strongly positively (Correlation = 0.643; P = 0.005) correlated with temperature index (Fig. 4A), whereas in this scenario it suggested RIR (Eigenvalue = − 0.1728), WRR (Eigenvalue = − 0.1542) and RS (Eigenvalue = − 0.1192) chickens should be best-adapted to cold. Besides, population eigenvalues of the Chinese indigenous in PC7 (1.98% variance totally explained) and PC6 (2.07% variance totally explained) were found to be moderately positively (Correlation = 0.511; P = 0.036) and negatively (Correlation = − 0.445; P = 0.076) correlated with temperature index (Fig. 4B,C) respectively. Similarly, WRR (Eigenvalue = − 0.0282) and RS (Eigenvalue = 0.2955) chickens would separately be the best-adapted to cold in these two scenarios. Considering that, a potential widespread genetic introgression from commercial chickens to Chinese indigenous high-latitude chickens has been observed and it will be hard to conclude that the cold-related variation to be identified from the Chinese indigenous chickens inhabiting in extreme temperature in winter is not because of genetic introgression from commercial chickens.

### Selective signatures in gamecock chickens

**Figure 5:**
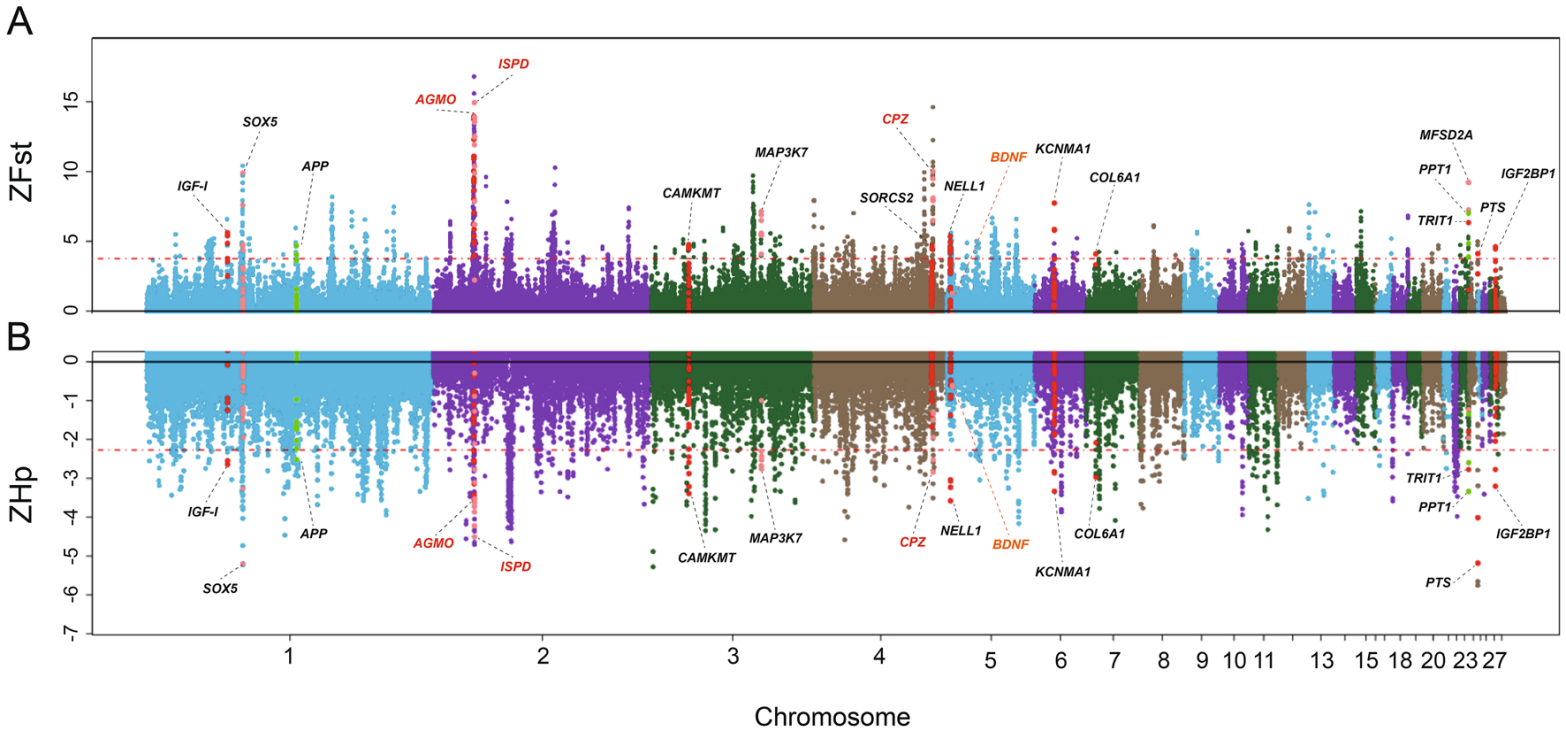
Genome-wide putatively selective signatures in the Gamecocks population. (**A**) Manhattan plot for each window calculated by ZFst. (**B**) Manhattan plot for each bin calculated by ZHp. *ISPD*, *AGMO* and *CPZ*, as the three genes with strongest selective signals are colored with red. *BDNF* as an important candidate selective gene concerning gamecock behavior proposed by Guo et al.^11^, is colored with orange on Chromosome 5.

After removing 198 and 1,326 windows with SNP number < 5, 92,010 and 91,940 were retained in subsequent statistics of Fst values and Hp scores respectively. With the threshold of top 1% outliers of windows being the putatively selective genomic regions, we identified 920 genomic regions in both ZFst (threshold score > 3.925) and ZHp (threshold score < − 2.251) analyses. This threshold proved to be robust enough to detect the genomic regions under selection in gamecock chickens after checking the distributions of ZFst value and ZHp score of each window along the autosomes (Figure S6). We further annotated the candidate genomic regions above, allowing us to identify 169 and 165 candidate genes in terms of ZFst and ZHp analyses respectively (Tables S6 & S7). However, only 31 genes were shared by both ZFst and ZHp analyses (Figure S7). In a previous report by Guo et al. concerning selective signatures in BN gamecock chickens^11^, which was also based on Fst (BN_vs_RJF) and Hp (within BN population) analyses (threshold: top 5% outliers), 343 candidate genes were identified (Table S8). While in the present study, we could just re-identify only 53 genes out of the earlier reported 343 genes (Figure S7), indicating that most of the selective genes previously identified were possibly the common ones by artificial selection during chicken domestication or biased by genetic drift. For instance, *CBFB*, *GRHL3*, *Gli3*, *BDNF*, *NTS*, *GNAO1* and *SDHB* as seven highlighted autosomal candidate selective genes were previously identified. Here, we just detected strong selective signals in *Gli3* (Table S9). Especially for *BDNF*, a gene involving the nervous system and aggressive behavior^21,22^, its selective signals in our study were very weak (Fig. 5). Further gene function annotation on the putatively selected genes from ZFst showed no biological processes (BPs) or KEGG pathways could be significantly enriched in, while for those from ZHp, several candidate genes could be significantly enriched in several BPs, including regulation of localization, regulation of cell migration, regulation of transport, regulation of cell motility, and positive regulation of NIK/NF-kappaB signaling (Table S10). In particular, candidate selective genes, including *APP*, *EGFR*, *MAP3K7*, *TCIM*, *CALR*, could be significantly (Adjusted *P value* = 0.049) enriched in positive regulation of NIK/NF-kappaB signaling, which concerns any process that activates or increases the frequency, rate or extent of NIK/NF-kappaB signaling. Importantly, NIK/NF-kappaB signaling is closely associated with immunity, and its loss function can induce a primary immunodeficiency with multifaceted aberrant lymphoid immunity^23^. These candidate selective genes may be conducive to the inflammation control of gamecock chickens in the context of their frequent fighting.

**Figure 6:**
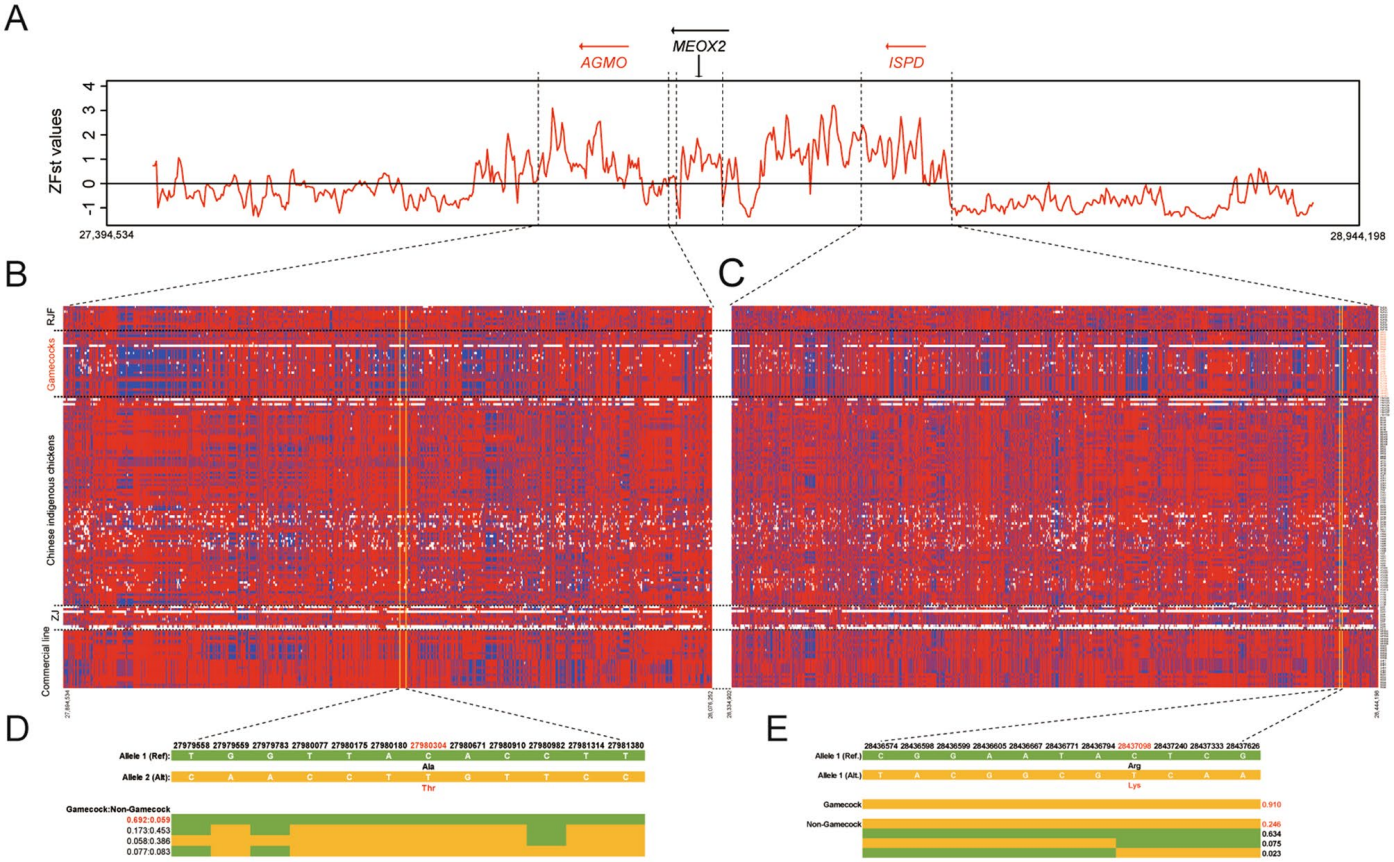
Selective sweeping signatures of *AGMO* and *ISPD* in gamecock chickens. (**A**) ZFst analysis of *AGMO* and *ISPD* between Gamecocks population and the other chickens except for RJF, with a window size of 5 Kb and a step size of 2 Kb employed. (**B**) Haplotype diversity of *AGMO* across 157 chickens, along with the structure of the block containing a non-synonymous SNP site Chr2:27,980,304 corresponding to Ala312Thr (ENSGALT00000037945.3) presented in (**D**). (**C**) Haplotype diversity of *ISPD* across 157 chickens, with the structure of the block containing a non-synonymous SNP site Chr2:28,437,098 corresponding to Arg84Lys (ENSGALT00000017557.5) given in (**E**). In both (**B**) and (**C**), the major and minor alleles of each site are separately colored with the red and the blue, while the white denotes the missing allele.

In this study, the sweeping loci with the highest ZFst values and much lower ZHp scores were observed from Chromosome 2:27,910,001–28,410,000 (Fig. 5A,B), within which *AGMO* (*Alkylglycerol monooxygenase*), *MEOX2* (*Mesenchyme homeobox 2*), and *ISPD* (*Isoprenoid synthase domain containing*) could be further identified (Fig. 6A). Only *AGMO* and *ISPD* exhibited a moderate level of linkage disequilibrium between each other (Figure S8), and two shared long-range haplotypes across gamecock chickens could be observed in *AGMO* (Fig. 6B) and *ISPD* (Fig. 6C), respectively, suggestive of strong sweeps of these two genes in gamecock chickens. Among those SNPs detected across the genomic regions of *AGMO* and *ISPD*, there were two possibly damaging and three probably benign missense mutations (Table S11). Particularly, the possibly damaging missense mutation (p.Ala312Thr) in exon 8 of *AGMO* was nearly fixed in non-gamecock chickens (fixation degree = 92.2%), but much less fixed in gamecock chickens (fixation degree = 30.2%) (Fig. 6D). While, the probably benign missense mutation (p.Arg84Lys) in exon 2 of *ISPD* was likely to be highly selected and favored in gamecock chickens, with a fixation degree reaching 91.0% (Fig. 6E). Further, conservativeness analysis of ISPD amino acid sequence across all 38 available avian species showed that the missense mutation (p.Arg84Lys) was conservative in birds and the missense Lys (K) detected in gamecock chickens could be just detected in Common Ostrich and American Crow (Figure S9). Interestingly, ostrich is the fastest living bipedal runner and possesses a muscular pelvic limb^24^.

As the only enzyme known to cleave the O-alkyl bonds of ether lipids (alkylglycerols), the missense variants of *AGMO* can induce microcephaly and neurodevelopmental disabilities in human beings^25,26^. For *ISPD*, it encodes a 2-C-methyl-d-erythritol 4-phosphate cytidylyltransferase-like protein, and it is essential for the glycosylation of α-dystroglycan in fibroblasts^27,28^. ISPD overexpression can independently or act synergistically with ribitol to improve dystrophic phenotype^29^. Its loss-of-function mutations can disrupt dystroglycan O-mannosylation, causing Walker-Warburg syndrome, which is defined as congenital muscular dystrophy and accompanied by a variety of brain and eye malformations^30^. Considering reasonably the above results together allow us to propose the variations in *AGMO*, and *ISPD* may play important roles in shaping the behavioral and muscular signatures of gamecock chickens observed respectively. Especially, the selective *ISPD* missense mutation of Arg84Lys (ENSGALT00000017557.5) in gamecock chickens, is possibly advantageous for the muscular development of gamecock chickens.

**Figure 7:**
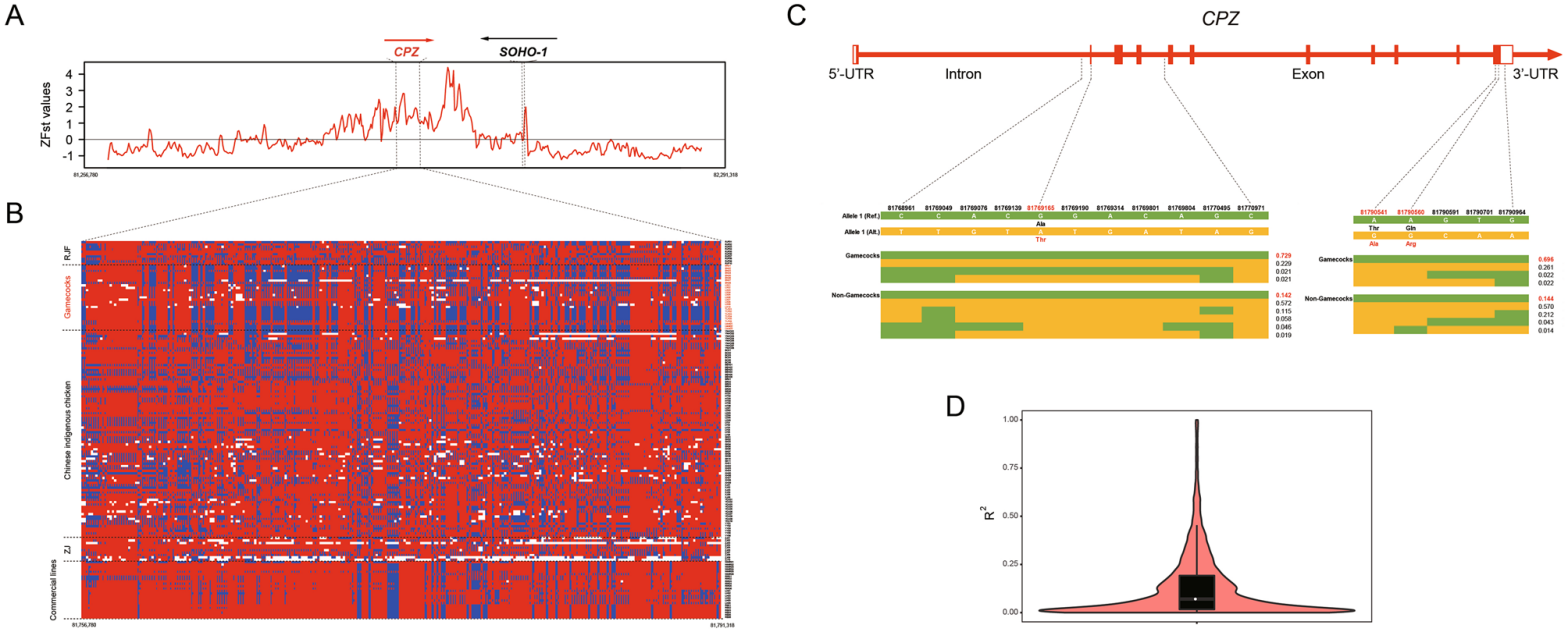
Selective sweeping signatures of *CPZ* in the Gamecocks population. (**A**) ZFst analysis of *CPZ* between Gamecocks population and the other chickens except for RJF, with a window size of 5 Kb and a step size of 2 Kb employed. (**B**) Haplotype diversity of *CPZ* across 157 chickens, inside which major and minor alleles of each site are separately colored with red and blue, and the missing allele is denoted with white. (**C**) The structures of two haplotype blocks within *CPZ* (ENSGALT00000061562.1) containing non-synonymous SNPs from Exon 2 and Exon 11. (**D**) The square of correlation coefficient (R^2^) regarding linkage disequilibrium between SNPs from the genomic region of *CPZ* (Chr4:81,756,785–81,791,318) and its adjacent downstream genomic region (Chr4:81,840,001–81,873,000) with higher genetic differentiation.

To further identify the genomic regions under selection in gamecock chickens concerning aggressive behavior, we mapped the candidate selective genomic regions in gamecock chickens to the chicken aggressive behavior quantitative trait loci (QTL) database (https://www.animalgenome.org/cgi-bin/QTLdb/GG/traitmap?trait_ID=2402). Thus, we discovered that the genomic region covering *CPZ* (*Carboxypeptidase Z*) was the only common one, for which it has been identified to be significantly associated with chicken fighting times^31^, and missense mutation within this gene can induce neuroblastoma in human beings^32^. Further haplotype homozygosity pattern analysis of genomic region covering *CPZ* across 157 chickens showed that a long-range haplotype was shared by gamecock chickens compared with RJF, Chinese indigenous and commercial chickens, suggestive of a strong selective sweep of this region (Fig. 7A,B). Additionally, we also identified three missense mutations from *CPZ* genomic region across 157 chickens, one from exon 2 (p.Ala34Thr) and two from exon 11 (p.Thr610Ala; p.Gln616Arg), with fixation degrees reaching 72.9% and 69.6% from exon 1 and exon 2 in gamecock chickens respectively (Fig. 7C). However, we could not exclude the hitchhiking effect on *CPZ* selection exerted by a downstream genomic region of Chr4:81,840,001–81,873,000. This downstream genomic region of *CPZ* harbored the highest genetic differentiation between gamecock chickens and others, within 500-kb upstream and downstream genomic regions of *CPZ* (Fig. 7A). Further LD analysis on those SNPs between *CPZ* (n = 247) and it is a downstream highly differentiated genomic region (n = 98) revealed some SNPs from these two genomic regions were at a high level of linkage disequilibrium (Fig. 7D). Collectively, these results above indicate that the variations in *CPZ* probably involved the aggressive behavior observed in gamecock chickens.

Furthermore, we could also identify *SOX5* (SRY-box 5), *NELL1* (*Neural EGFL like 1*), *KCNMA1* (*Potassium calcium-activated channel subfamily M alpha 1*), *IGF-I* (*Insulin like growth factor 1*) and *IGF2BP1* (*Insulin like growth factor 2 mRNA binding protein 1*), harbored strikingly higher ZFst values and/or lower ZHp scores (Fig. 4; Table 2), suggestive of strong selective sweeps of these genes in gamecock chickens. Except for *IGF2BP1*, another above five genes were previously reported by Guo et al. as well^11^, highlighting their selective sweeping consistency in gamecock chickens. Among them, *SOX5* has proved to be the causative gene underlying pea-comb in chicken^33^, probably explaining the pea-comb phenotype commonly observed in Chinese gamecock chickens. *IGF2BP1*, a gene closely associated with body size and growth in ducks^34^, together with the previously reported *IGF-I*, they probably have played important roles in determining the large body size of gamecock chickens.

**Table 2:** Major selective genes concerning the morphological, physiological and behavioral signatures in Gamecocks, identified from both ZFst and ZHp.

Gene ID	Chromosome region (Galgal 5.0)	Max ZFst value	Min ZHp score	Function related
*IGF-I*	Chr1:55,335,204–55,383,631	5.62	− 2.64	Body size^35^
*SOX5*	Chr1:65,938,356–66,226,982	9.90	− 5.20	Pea-comb^33^
*APP*	Chr1:102,609,866–102,809,834	4.69	− 2.52	Autistic behavior and aggression^36^
*AGMO*	Chr2:27,894,534–28,076,252	13.97	− 3.55	Syndromic microcephaly and neurodevelopmental disorder^25,26^
*ISPD*	Chr2:28,334,902–28,444,198	14.95	− 4.51	Muscular strength^29^
*CPZ*	Chr4:81,756,785–81,791,318	10.01	− 2.84	Aggressive behavior and neurodevelopment^31,32^
*NELL1*	Chr5:2,201,059–2,481,132	5.35	− 3.58	Bone formation^37^
*KCNMA1*	Chr6:13,362,771–13,800,472	7.74	− 3.33	Osteoblast bone formation^38^
*COL6A1*	Chr7:6,723,053–6,744,124	4.10	− 2.97	Muscular strength^39^
*MFSD2A*	Chr23:5,554,873–5,562,608	9.22	− 3.34	Normal brain growth and cognitive function^40^
*IGF2BP1*	Chr27:3,648,588–4,003,342	4.61	− 3.21	Body size and feed efficiency^34^

Max ZFst value and Min ZHp score of each gene here are denoted with the windows from the corresponding gene that harbor the highest ZFst value and the lowest ZHp score, respectively.

## Conclusions

In conclusion, we here characterized the genome diversity, linkage disequilibrium pattern, genetic differentiation, population structure and migration events, across the 157 chickens (126 ones sequenced here) from 22 populations, and re-identified the selective signatures in gamecock chickens with potential confounding effects exerted by introgression and genetic drift fully considered. Our results showed that the Chinese indigenous chickens except those breeds having muffs and beard phenotype were less intensively selected, and a widespread introgression from commercial chickens into them might have occurred, for which it could have severely confounded the selection footprints in indigenous chickens, such as cold adaptation. Importantly, we identified *AGMO* and *CPZ* might be crucial for determining the behavioral pattern, while *ISPD* might be essential for the muscularity observed in gamecock chickens. These results together can facilitate conservation of the 13 canonical Chinese indigenous breeds, and the genetic basis of gamecock chickens revealed here is valuable for us to better understand the mechanisms underlying the behavioral pattern and the muscular development in chicken.

## Materials and methods

### Sampling and genome sequencing

A total of 126 blood samples from 19 chicken populations were collected from 19 populations which composed of 13 Chinese nationwide indigenous chicken breeds, including six Huiyang Bearded chickens (BC), nine Xinghua chickens (XH), six Hetian chickens (HT), six Baier Yellow chickens (BEH), 11 Silkies (SK), six Xianju chickens (XJ), six Liyang chickens (LY), six Jining Bairi chickens (BR), six Yunyang Da chickens (YY), ten Beijing You chickens (YOU), six Lindian chickens (LD), ten Luxi gamecock chickens (LX) and six Tulufan gamecock chickens (TLF) (Fig. 1A); four typical commercial populations, including six White Leghorn chickens (LH), six White Recessive Rocks (WRR), six Cobb RS308 chickens (RS) and six Rhode Island Reds (RIR); one Red jungle fowl population from Guangxi Province (five individuals, RJF) and one gamecock population from Laos (three individuals, Laos). Genomic DNA was further extracted from the collected blood samples using NRBC Blood DNA Kit (Omega Bio-Tek, Norcross, GA, USA) following the manufacturer’s instruction, and the quality of the extracted Genomic DNA was tested using Nanodrop 2000 spectrophotometer at 260/280 nm ratio (NanoDrop Inc., Wilmington, DE, USA). To provide a more comprehensive understanding and profound insight into the genome diversity of Chinese indigenous chickens and the genetic base underlying Chinese gamecock chickens, we incorporated the sequencing data of another eight Xishuangbanna gamecock chickens (BN), eight Yunnan village chickens (YNVC), ten Tibetan chickens (ZJ) and five Red jungle fowls (RJF), which has been previously published^4^. Overall, we generated a panel of 157 miscellaneous chickens, which were from 22 populations. These 157 chickens from 22 populations could be further grouped into eight categories, including Low-latitude (BC, XH, and YNVC), Middle-latitude (HT, BEH, XJ, SK, BR, YY and LY), High-latitude (LD and YOU), High-altitude (ZJ), Gamecocks (Laos, TLF, LX, and BN), Commercial broilers (WRR and RS), Commercial layers (RIR and LH) and Ancestry (RJF) (Note S1; Table S1).

More than 3 μg of genomic DNA from the above samples were used to construct a paired-end sequencing library with an insert size of approximately 350 bp following the manufacturer’s instructions, thereby being sequenced on Illumina HiSeq X Ten and HiSeq 2000 platforms (Illumina, San Diego, CA, USA) at Novogene Co., Ltd (Beijing, China) and Beijing Institute of Genomics (Beijing, China). After removing the sequencing paired-end reads with adaptors, N content ratio > 10% and low-quality base ratio (Q ≤ 5) > 50%, clean reads were retained for subsequent genome mapping and variant calling.

### Genome mapping, variant calling and annotation

Firstly, we used Burrows-Wheeler Aligner (BWA) version 0.7.15 to map the clean sequencing reads to the Gallus gallus 5.0 reference genome (https://www.ncbi.nlm.nih.gov/assembly/GCF_000002315.4/)^41^, generating the Sam file for each sample. SAMtools version 1.3^42^, was then used to filter out the unmapped and non-unique reads from the above Sam files following the command “rmdup” and generate the corresponding BAM format files. Meanwhile, Picard version 2.9.0 (https://broadinstitute.github.io/picard/) was employed to sort the SAM files into coordinate order and further saved as binary alignment map files (BAM files), followed with duplicate reads marked and BAM files indexed. We here utilized SAMtools version 1.3 and GATK version 3.7.0^43^, simultaneously to detect SNP and InDel at a population level, only with the SNPs and InDels detected by both pipelines kept for further analysis. For SAMtools calling, raw SNPs and raw InDels were called using the SAMtools mpileup package with default parameters. Before GATK calling, we performed a step of base quality score recalibration to get more accurate base qualities, in which a set of over 14 million known chicken SNP data from Ensembl database (ftp://ftp.ensembl.org/pub/release-94/variation/gvf/gallus_gallus/) was used together with GATK version 3.7.0 “BaseRecalibrator” to generate the recalibrated BAM files. Further, the engine “Unifiedgenotyper” in GATK (default settings) was employed to call the raw SNPs and InDels. Finally, the common sites of SNP/InDel identified by both SAMtools and GATK were retained, and the SNPs were further submitted to VCFtools version 0.1.14^44^, for quality control using the following filtration criteria: (1) max-missing 0.1; (2)—maf 0.05; (3)—minQ 20; (4)—min-meanDP 5; (5) max-meanDP 1,000; (6)—minGQ 5, in which the SNPs and InDels sites with missing data < 0.1, minor allele frequency (MAF) > 0.05, quality value > 20, mean depth values between 5 and 1,000, and genotype quality above 5 were kept for subsequent analyses. To annotate the SNPs and InDels identified here, ANNOVAR (Version: 2013-05-20) was employed^45^. Considering our samples which consisted of males and females, we further extracted the autosomal SNPs for genetic differentiation, pooled-heterozygosity, LD, population genetic structure and selective sweep analyses at the genome-wide level to avoid non-stochastic effects.

### Genome-wide nucleotide diversity and heterozygosity, linkage disequilibrium

We herein assessed the genome diversity by calculating the genome-wide nucleotide diversity and pooled-heterozygosity within each population. Genome-wide nucleotide diversity ($\pi$) within each population was measured in windows using VCFtools version 0.1.14^44^, with a window size of 40 Kb and a step size of 20 Kb. For the pooled-heterozygosity (Hp) within each population, we calculated the pooled-heterozygosity score of each window (Window size: 40 Kb; Step size: 20 Kb) following the formula given by Rubin et al.^46^:

$$Hp = 2\sum nMAJ\sum nMin/\left( {\sum nMAJ + \sum nMin} \right)^{2} .$$

Haploview version 4.2^47^, was used to evaluate the genome-wide linkage disequilibrium pattern within each population, with arguments “—maxdistance 500;—minMAF 0.05;—binsize 100” employed. Also, it was utilized to infer the square of the correlation coefficient (r^2^), haplotype structure and frequency for some specific genomic regions presented in this study.

Considering the Laos gamecock population that had only three individuals here, we didn’t consider its related results in terms of the above analyses. The genome-wide nucleotide diversity ($pop\_\pi$) and the heterozygosity of each population ($pop\_He$) were measured with the mean values of all windows’ $\pi$ and *Hp*.

### Genetic differentiation

For the genetic differentiation between each population, VCFtools version 0.1.14 was used to calculate the pairwise Fst values between each population^48^, with a window size of 40 Kb and a step size of 20 Kb.

### Population genetics analysis

We used TreeBeST version 1.9.2 software^49^, to calculate the distance matrix and thus constructed a neighbor-joining tree (bootstrap values = 1,000) with all identified autosomal SNPs. Before performing the Principal component analysis (PCA) and Admixture analysis, all population autosomal SNPs were firstly LD-based pruned using Plink version 1.9^50^, (https://pngu.mgh.harvard.edu/purcell/plink/) with the option “indep-pairwise 50 5 0.5” employed. Based on the pruned population SNP data, we then performed Principal component analysis (PCA) and unsupervised Admixture analysis to assess the population’s genetic structure. For the PCA, smartpca program in Eigenstrat version 6.1.4^51^, was adopted with the explained variance given in according to its corresponding eigenvalue proportion in the sum of eigenvalues. Meanwhile, Admixture version 1.3.0^52^, was run with K = 2 to K = 16, along with their corresponding cross-validation errors (default setting used) calculated, respectively.

To estimate the potential impact exerted by extreme temperature in winter on Chinese indigenous chickens, mean eigenvalues of each population (population eigenvalue) at each principal component (PC) were calculated. Pearson correlation analysis was then performed between the extreme temperature of each Chinese indigenous population and its corresponding population eigenvalue at each PC.

### TreeMix analysis

We used TreeMix software^53^, to infer the historical relationships of the 22 chicken populations included here. We ran TreeMix with migration events given from 1 to 10, and generated their corresponding residual matrix, with options “-noss” and “-k 500” used. A tree with the smallest residuals was to be the best fit for the data. Considering the wild population (RJF chickens) included here could not be grouped into the same cluster in the phylogenetic analysis, we did not root the maximum likelihood tree.

### Genome-wide selective sweep analysis

We employed two methods here, including calculating the genetic differentiation (Fst) between gamecock chickens (TLF, LX, Laos, and BN chickens) and non-gamecocks (chickens except RJF and gamecock chickens) and the pooled-heterozygosity score (Hp) within gamecock chickens upon sliding windows, to identify the genomic regions under selection in gamecocks population. Considering the gamecock populations harbor relatively high genome nucleotide diversity and heterozygosity in chickens, we narrowed down the window size and step size to 20 Kb and 10 Kb when calculating both Weir-Fst value and Hp score of each window. We eliminated the windows with SNPs less than 5 to ensure detective accuracy. The top 1% outliers of bins were regarded as the putative genomic regions under selection, and further annotated using Ensembl BioMart tool (https://oct2018.archive.ensembl.org/biomart/martview/fcee6700cde0db959bc30ef4fc9d839a). Those putatively selected genes from each method were then submitted to gProfiler (https://biit.cs.ut.ee/gprofiler/gost) for function enrichment analysis with options “Organism: Gallus gallus” and “User threshold: 0.05”. Both the Fst value and Pooled-heterozygosity score of each bin were Z-transformed according to the formula below and further Manhattan-plotted with in-house R scripts:

$$ \begin{aligned} ZFst & = {{(Fst - \mu Fst)} \mathord{\left/ {\vphantom {{(Fst - \mu Fst)} {\sigma Fst}}} \right. \kern-\nulldelimiterspace} {\sigma Fst}}, \\ ZHp & = {{(Hp - \mu Hp)} \mathord{\left/ {\vphantom {{(Hp - \mu Hp)} {\sigma Hp}}} \right. \kern-\nulldelimiterspace} {\sigma Hp}}. \\ \end{aligned} $$

PANTHER version 11.0 (https://www.pantherdb.org/tools/csnpScoreForm.jsp?)^54^, was employed to estimate the likelihood of nonsynonymous (amino-acid changing) coding SNPs to cause a functional impact on the proteins of ISPD, AGMO, and CPZ.

### Research ethics statement and data availability

All the animal experiments used in the present study were approved by the South China Agricultural University Institutional Animal Care and Use Committee (Approval number: 2015-A003; Guangzhou, People’s Republic of China), and were handled strictly in compliance with the guidelines of this committee.

The genome sequencing raw data has been uploaded into the NCBI SRA database with the accession number SAMN14651083.

## Supplementary information

Supplementary Information.

Supplementary Figures.

Supplementary Tables.
