# Supplementary material for: Genome diversity of Chinese indigenous chicken and the selective signatures in Chinese gamecock chicken
Source: Sci Rep. 2020 Sep 3;10:14532. doi: 10.1038/s41598-020-71421-z (PMC7471287; doi:10.1038/s41598-020-71421-z)
Supplement: Supplementary file 1 — Supplementary Information. [file 41598_2020_71421_MOESM1_ESM.docx]

Genome diversity of Chinese indigenous chicken and the selective signatures in Chinese gamecock chicken

**Wei Luo^1,3#^, Chenglong Luo^2#^,** **Meng Wang^4^, Lijin Guo^1,3^, Xiaolan Chen^1,3^, Zhenhui Li^1,3^, Ming Zheng^1,3^, Bello Semiu Folaniyi^1,3^, Wen Luo^1,3^, Linliang Song^5^, Meixia Fang^5^, Dingming Shu^2^, Xiquan Zhang^1,3^, Hao Qu^2*^, Qinghua Nie^1,3*^**

^1^ Department of Animal Genetics, Breeding and Reproduction, College of Animal Science, Guangzhou 510642, Guangdong, China;

^2^ State Key Laboratory of Livestock and Poultry Breeding & Guangdong Key Laboratory of Animal Breeding and Nutrition, Institute of Animal Science, Guangdong Academy of Agricultural Sciences, Guangzhou 510640, China;

^3^ Guangdong Provincial Key Lab of Agro-Animal Genomics and Molecular Breeding and Key Lab of Chicken Genetics, Breeding and Reproduction, Ministry of Agriculture, Guangzhou 510642, Guangdong, China;

^4^ Novogene Bioinformatics Institute, Beijing, China;

^5^ Institute of Laboratory Animals, Jinan University, Guangzhou, Guangdong, China.

***** Correspondence: Qinghua Nie, e-mail: [nqinghua@scau.edu.cn](mailto:nqinghua@scau.edu.cn); Hao Qu, email: [qhw03@163.com](mailto:qhw03@163.com)

**^#^** Authors to whom contribute equally to this work.

**Note S1.** Characteristics of the Chinese indigenous and commercial breeds/populations used in this study.

***Ancestry:***

**RJF** (red jungle fowl) chicken consists of 5 subspecies, including *G. g. gallus*, *G. g. jabouillei*, *G. g. spadiceus*, *G. g. murgha*, and *G. g. bankiva*^1^. It is the main ancestry of domestic chickens^1,2^. In China, it is mainly distributed in provinces of Yunnan, Guangdong and Hainan, and Guangxi Zhuang Autonomous Region. We here sampled and sequenced 5 RJF chickens (RJF06-RJF10) from Lingshan County, Guangxi Zhuang Autonomous Region, which seem to be *G. g. spadiceus*. Additionally, we also incorporated the genome data of another 5 public RJF chickens, which were sampled from Yunnan Province^3^.

***Gamecock chickens:***

China has a long history in the utility of chicken cockfighting. There is a total of 6 officially recorded gamecock chicken breeds in China, including **LX (Luxi Gamecock chicken; Originating from Southwest Shandong Province)**, HN (Henan Gamecock chicken; Originating from Kaifeng City, Henan Province), **TLF (Tulufan Gamecock** chicken**; Originating from Turpan Basin, Xinjiang Uygur Autonomous Region)**, **BN (Xishuangbanna Gamecock** chicken**; Originating from Xishuangbanna, Yunnan Province)**, WB (Wanbei Gamecock chicken; Originating from Bozhou City, Anhui Province) and ZZ (Zhangzhou Gamecock chicken; Zhangzhou City). Of these 6 gamecock breeds, **LX** and HN gamecock breeds are the earliest recorded, which can be dated back to 2,700 BC^4^. Chinese gamecock chickens are commonly featured with their muscularity and strong aggressiveness, and have a larger body size compared with most Chinese indigenous chicken breeds.^3^ We here sampled and sequenced 10 **LX** and 6 **TLF** gamecock chickens, and incorporated the genome data of another 8 public **BN** gamecock chickens^3^. Additionally, to better understand the mechanisms underlying the signatures of gamecock chickens, 3 gamecock chickens from **Laos** were sampled and sequenced.

***High-latitude Chinese indigenous chickens:***

**LD** chicken (**Lindian** chicken), used for both meat and egg productions, is a Chinese indigenous breed, which originates from Liandian County, Heilongjiang Province. This chicken has evolutionarily adapted to the extreme cold winter since imported from North China in the early 19^th^ century;^4^ In this study, we sampled and sequenced 6 **LD** chickens.

**YOU** chicken (**Beijing You** chicken), used for both meat and egg productions, is a famous Chinese indigenous breed, which originates from Beijing City and mainly featured with the special phenotypes, such as crest, feathered shank, and muffs and beard.^4,5,6^ We here sampled and sequenced 10 **YOU** chickens.

***Middle-latitude Chinese indigenous chickens:***

**BR** chicken (**Jining Bairi** chicken), mainly used for egg production, is a Chinese indigenous breed. It originates from Jining City, Shandong Province, and featured with its early sexual maturity;^4^ 6 **BR** chickens were sampled and sequenced.

**YY** chicken (**Yunyang Da** chicken), used for both meat and egg productions, is a Chinese indigenous breed, which originates from Zhushan County, Hubei Province;^4^ We here sampled and sequenced 6 **YY** chickens.

**LY** chicken (**Liyang** chicken), mainly used for meat production, is a Chinese indigenous breed, originates from Liyang City, Jiangsu Province. This chicken is mainly featured with its large body size among Chinese indigenous chickens;^4^ 6 **LY** chickens were sampled and sequenced in this study.

**XJ** chicken (**Xianju** chicken), mainly used for egg production, is a Chinese indigenous breed and originates from Xianju County, Zhejiang Province. This chicken was first recorded in 1,600 AD. It is mainly featured with its high-performance egg production among Chinese indigenous chickens^4^; 6 **XJ** chickens were sampled and sequenced in this study.

**BEH** chicken (**Baier Yellow** chicken), mainly used for egg production, is a Chinese indigenous breed, originating from Guangfeng County, Jiangxi Province. It has white earlobes and high-performance egg production among Chinese indigenous chickens.^4^ We here sampled and sequenced 6 **BEH** chickens.

**SK** chicken (**Silkies**), used for both meat and egg production, is an old world-famous Chinese indigenous breed. This chicken was first recorded in the 13^th^ century and originates from several cities from Jiangxi and Fujian provinces. It is featured with a number of special external traits, such as crest, rose-comb, black-skin, green earlobe, muffs and beard, silky-feather, feathered-shank, and polydactyly phenotypes.^4,5,6,7,8,9,10^ 11 **SK** chickens were sampled and sequenced in this study.

**HT** chicken (**Hetian** chicken), mainly used for meat production, originates from Changting County, Fujian Province. This breed was first recorded in 1,752 AD. It is mainly featured with its unique comb phenotype that resembles antler.^4^ 6 **HT** chickens were sampled and sequenced in this study.

**BC** chicken (**Huiyang Bearded** chicken), mainly used for meat production, is an old Chinese indigenous breed that can be dated back to 1,700 years ago.^4^ This breed originates from counties of Guangdong Province like Huiyang, Boluo, Zijin, Longmen and Huidong. It is mainly featured with the muffs and beard phenotype.^6^ We here sampled and sequenced 6 **BC** chickens.

**ZJ** chicken (**Tibetan** chicken), used for both meat and egg productions, is a Chinese indigenous breed. This chicken resembles RJF and originates from Tibetan Plateau.^4^ It has evolutionarily adapted to the plateau environments such as hypoxia and cold.^3^ We here incorporated the public genome data of 10 ZJ chickens.^3^

**YNVC** chicken is Yunnan village chickens, which sequenced in a previous study.^3^ We here incorporated the public genome data of 10 YNVC chickens.

***Commercial chickens:***

**WRR** chicken (**White Recessive Rock** chicken)**, RS308** chicken (**Ross308** chicken)**, RIR** chicken (**Rhode Island Red** chicken)**,** and **LH** chicken (**White Leghorn** chicken), these four breeds are the commonly farmed commercial chickens in China, usually used for breeding materials. Of them, **LH** and **RIR** chickens harbor high-performance egg production, while WRR and RS308 are excellent for meat production. We here sampled and sequenced 6 chickens for each above commercial breed.

**Reference:**

1. Wang, M. S., Thakur, M., Peng, M. S., et al. 2020, 863 genomes reveal the origin and domestication of chicken, *Cell. Res.*, doi: 10.1038/s41422-020-0349-y. Online ahead of print.
2. Fumihito, A., Miyake, T., Takada, M., et al. 1996, Monophyletic origin and unique dispersal patterns of domestic fowls, *Proc. Natl. Acad. Sci. USA.*, 93(13), 6792-5.
3. Wang, M. S., Li, Y., Peng, M. S., et al. 2015, Genomic Analyses Reveal Potential Independent Adaptation to High Altitude in Tibetan Chickens, *Mol*. *Biol*. *Evol.*, 32(7), 1880-9.
4. Chen, K. W., Yang, N., Wang, G. Y., et al. 2011, Animal Genetic Resources in China: Poultry, 1st Ed., China National Commission of Animal Genetic Resources, China Agricultural Press, Beijing.
5. Wang, Y., Gao, Y., Imsland, F., et al. 2012, The crest phenotype in chicken is associated with ectopic expression of HOXC8 in cranial skin, *PLoS. One.*, 7(4), e34012.
6. Guo, Y., Gu, X., Sheng, Z., et al. 2016, A Complex Structural Variation on Chromosome 27 Leads to the Ectopic Expression of HOXB8 and the Muffs and Beard Phenotype in Chickens, *PLoS, Genet*., 12(6), e1006071.
7. Feng, C., Gao, Y., Dorshorst, B., et al. 2014, A cis-regulatory mutation of PDSS2 causes silky-feather in chickens, *PLoS. Genet.*, 10(8), e1004576.
8. Dorshorst, B., Molin, A. M., Rubin, C. J., et al. 2011, A complex genomic rearrangement involving the endothelin 3 locus causes dermal hyperpigmentation in the chicken, *PLoS. Genet.*, 7(12), e1002412.
9. Zhang, Z., Nie, C., Jia, Y., et al. 2016, Parallel Evolution of Polydactyly Traits in Chinese and European Chickens, *PLoS. One*., 11(2), e0149010.
10. Imsland, F., Feng, C., Boije, H., et al. 2012, The Rose-comb mutation in chickens constitutes a structural rearrangement causing both altered comb morphology and defective sperm motility, *PLoS. Genet.*, 8(6), e1002775.
