# Supplementary material for: Genome diversity of Chinese indigenous chicken and the selective signatures in Chinese gamecock chicken
Source: Sci Rep. 2020 Sep 3;10:14532. doi: 10.1038/s41598-020-71421-z (PMC7471287; doi:10.1038/s41598-020-71421-z)
Supplement: Supplementary file 2 — Supplementary Figures. [file 41598_2020_71421_MOESM2_ESM.docx]

Genome diversity of Chinese indigenous chicken and the selective signatures in Chinese gamecock chicken

**Wei Luo^1,3#^, Chenglong Luo^2#^,** **Meng Wang^4^, Lijin Guo^1,3^, Xiaolan Chen^1,3^, Zhenhui Li^1,3^, Ming Zheng^1,3^, Bello Semiu Folaniyi^1,3^, Wen Luo^1,3^, Linliang Song^5^, Meixia Fang^5^, Dingming Shu^2^, Xiquan Zhang^1,3^, Hao Qu^2*^, Qinghua Nie^1,3*^**

^1^ Department of Animal Genetics, Breeding and Reproduction, College of Animal Science, Guangzhou 510642, Guangdong, China;

^2^ State Key Laboratory of Livestock and Poultry Breeding & Guangdong Key Laboratory of Animal Breeding and Nutrition, Institute of Animal Science, Guangdong Academy of Agricultural Sciences, Guangzhou 510640, China;

^3^ Guangdong Provincial Key Lab of Agro-Animal Genomics and Molecular Breeding and Key Lab of Chicken Genetics, Breeding and Reproduction, Ministry of Agriculture, Guangzhou 510642, Guangdong, China;

^4^ Novogene Bioinformatics Institute, Beijing, China;

^5^ Institute of Laboratory Animals, Jinan University, Guangzhou, Guangdong, China.

***** Correspondence: Qinghua Nie, e-mail: [nqinghua@scau.edu.cn](mailto:nqinghua@scau.edu.cn); Hao Qu, email: [qhw03@163.com](mailto:qhw03@163.com)

**^#^** Authors to whom contribute equally to this work.


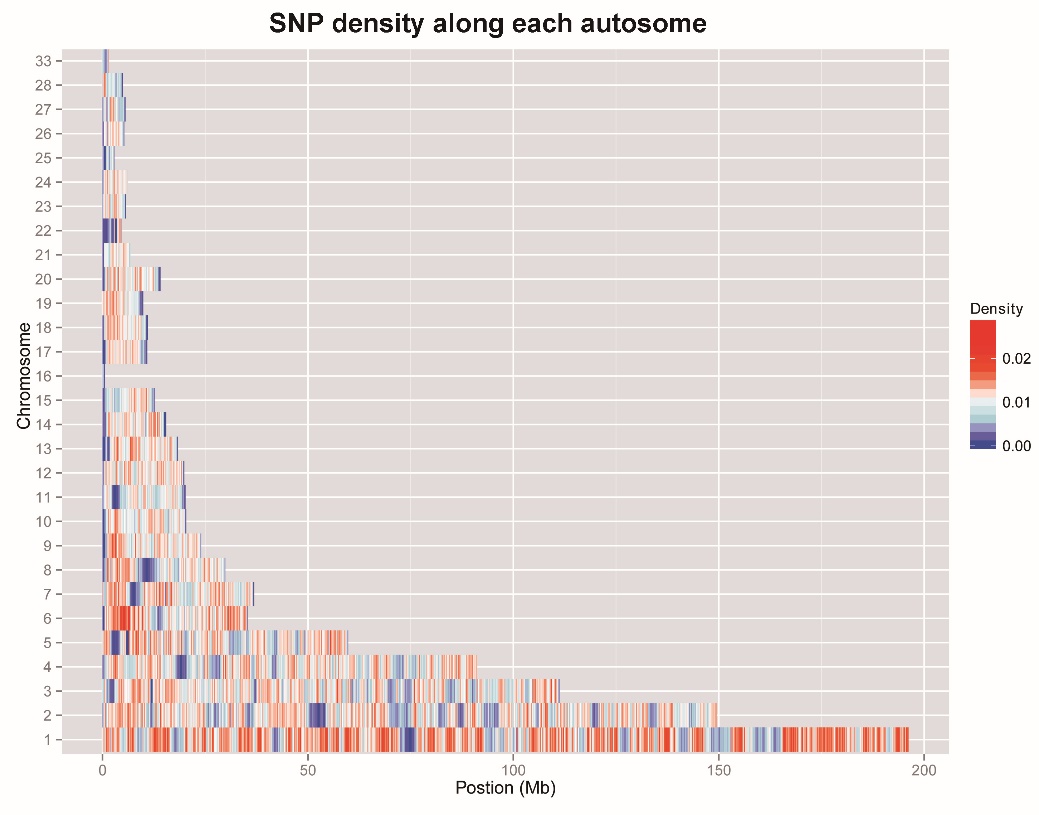


**Fig S1.** SNP density along each autosome, scaled by the proportion of SNPs within each 100-Kb genomic region.


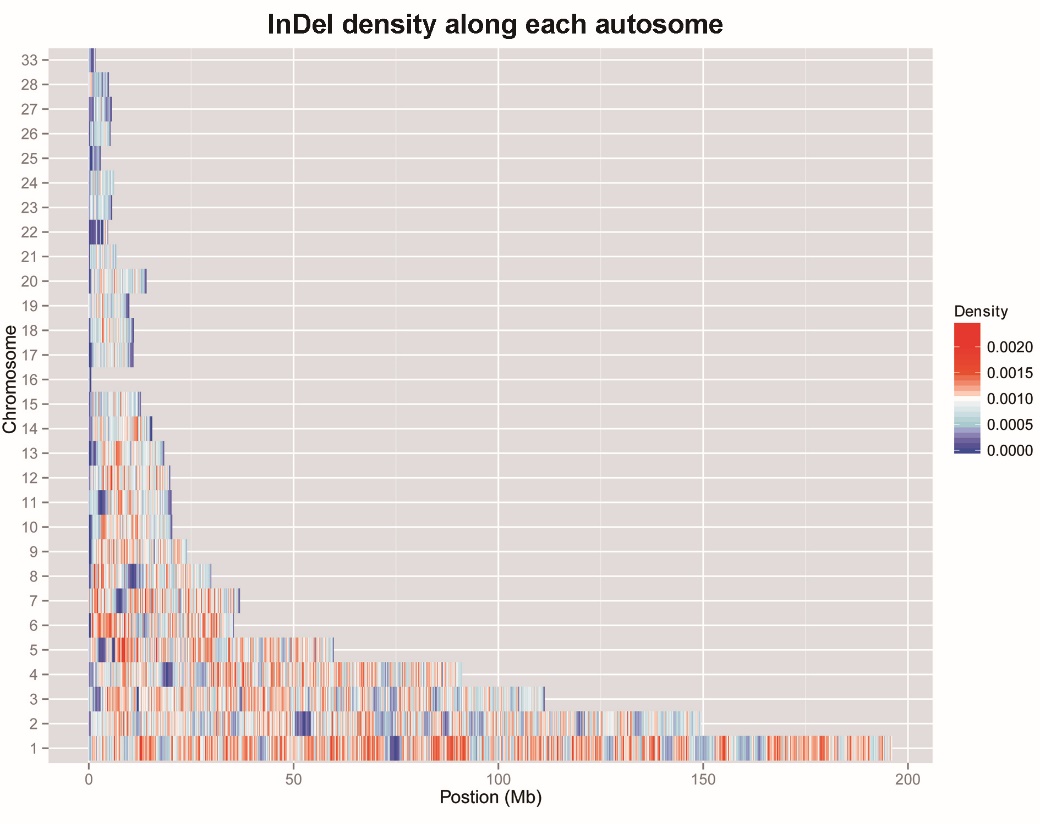


**Fig S2.** InDel density along each autosome, scaled by the proportion of InDels within each 100-Kb genomic region.


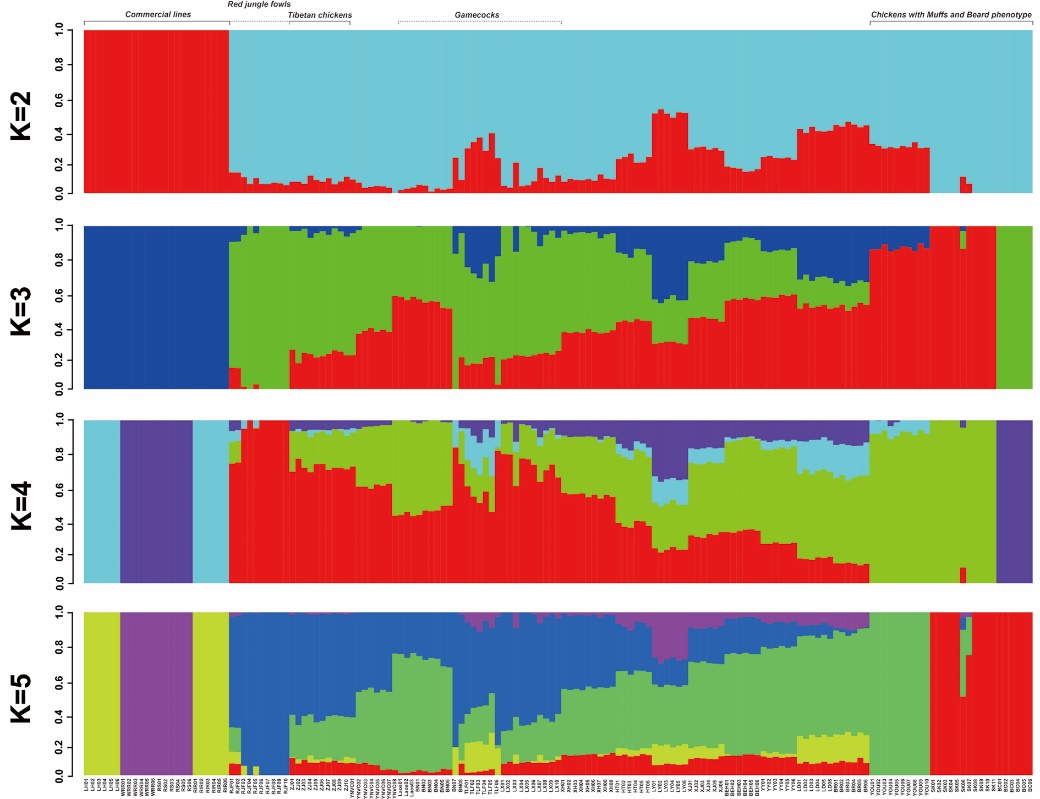


**Fig S3.** Admixture analysis with K run from 2 to 5. A potential widespread introgression from commercial chickens to most Chinese indigenous chicken breeds can be evidenced, except those three breeds (BC, SK and YOU chickens) with muffs and beard phenotype. Additionally, gamecock chickens are genetically likely to be the admixed by RJF, Chinese indigenous and commercial chickens.


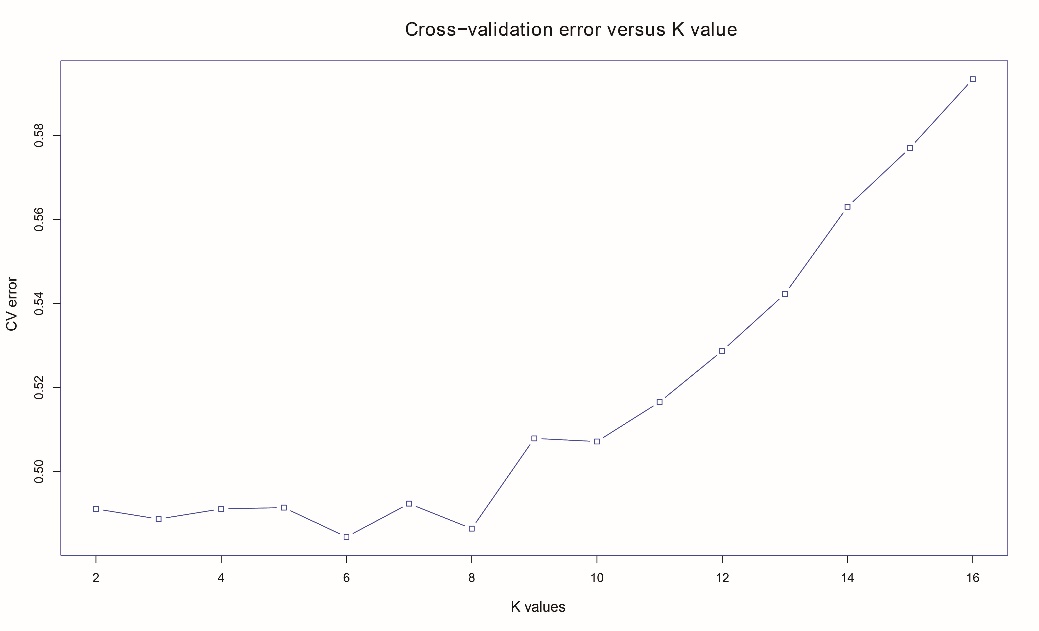


**Fig S4.** Cross-validation (CV) error returned from K = 2 to K = 16, in which the smallest CV error can be observed at K = 6.


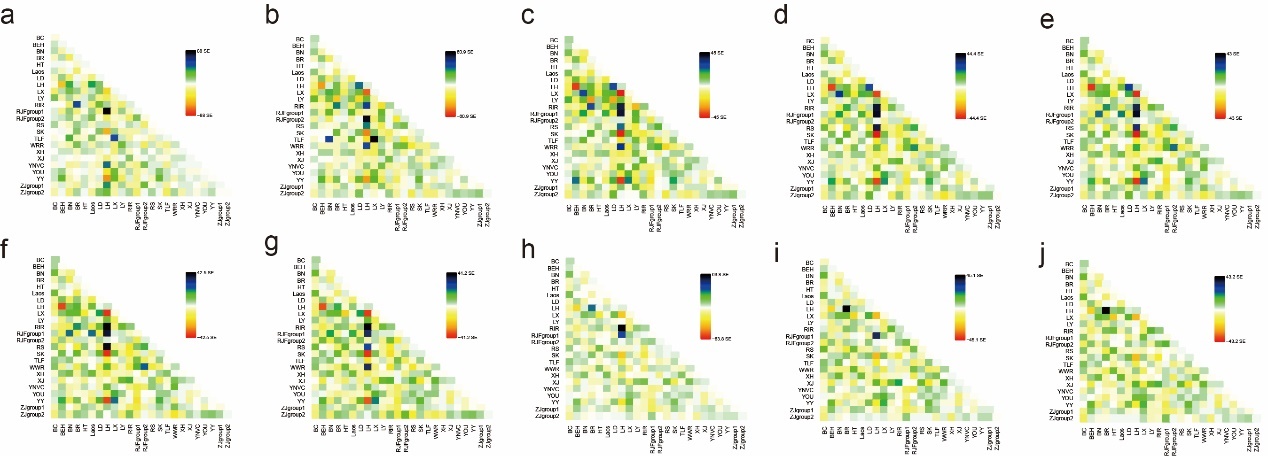


**Fig S5.** Residual matrix of each migration event scenario (from 1 to 10 migration events, corresponding to a-j, respectively), in which adding 7 migration edges show the smallest residuals.


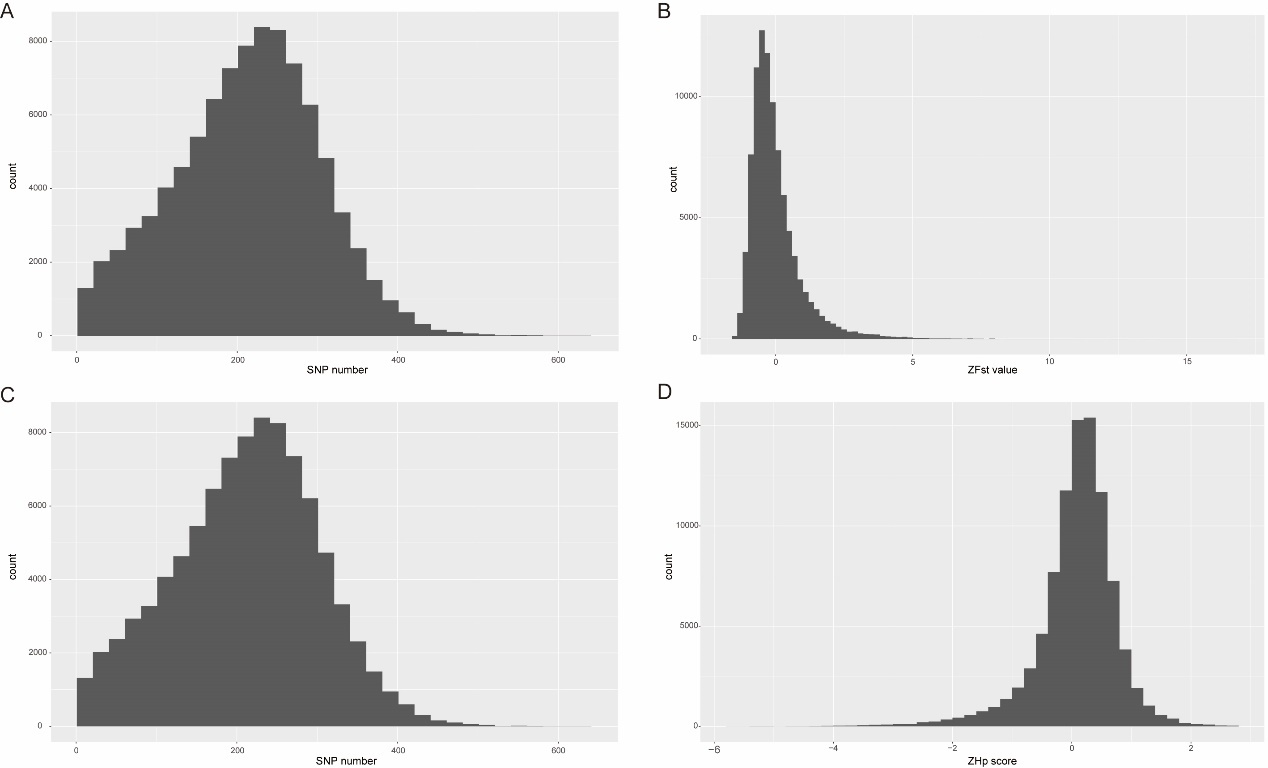


**Fig S6.** (A) Statistics of SNP number distribution of all chickens by a 20-Kb bin (window size = 20 Kb; step size = 10 Kb). (B) Statistics of ZFst values from each bin (window size = 20 Kb; step size = 10 Kb). (C) Statistics of SNP number distribution of gamecock chickens by a 20-Kb bin (window size = 20 Kb; step size = 10 Kb). (D) Statistics of ZHp scores from each bin (window size = 20 Kb; step size = 10 Kb).


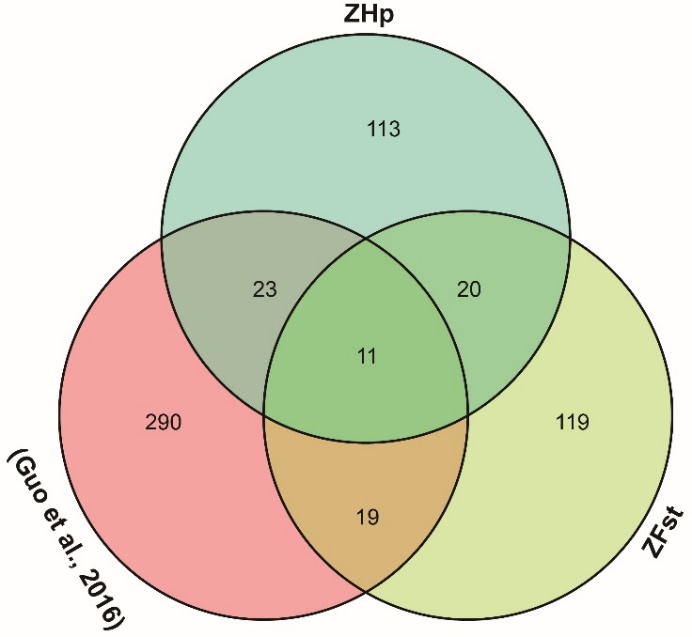


**Fig S7.** Veen diagram of the candidate selective genes identified in this study and by the study of Guo et al (2016).


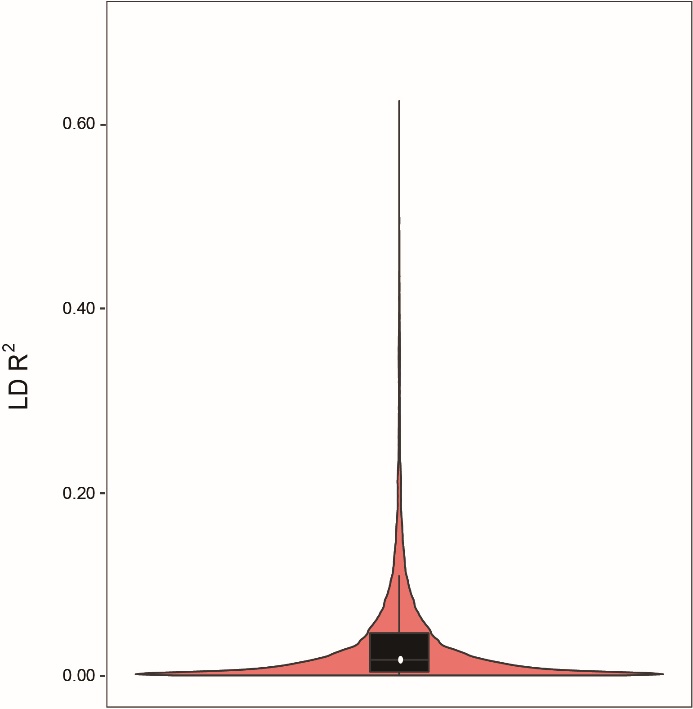


**Fig S8.** The square of correlation coefficient (R2) regarding linkage disequilibrium between SNPs from the genomic region of *AGMO* (Chr2:27,894,534-28,076,252) and *ISPD* (Chr2:28,334,902-28,444,198).


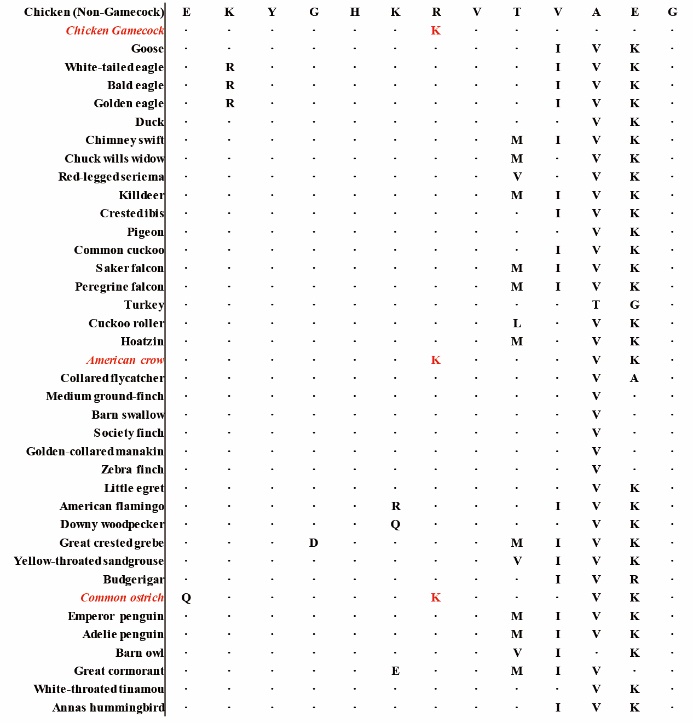


**Fig S9.** Conservativeness analysis of the missense mutation (p.Arg84Lys) of ISPD amino acid sequence across all 38 available avian species.
